# Hypertrophic Cardiomyopathy Phenocopies: Classification, Key Features, and Differential Diagnosis

**DOI:** 10.3390/biomedicines13123062

**Published:** 2025-12-12

**Authors:** Lucio Teresi, Giancarlo Trimarchi, Roberto Licordari, Davide Restelli, Giovanni Taverna, Paolo Liotta, Antonino Micari, Ignazio Smecca, Gregory Dendramis, Dario Turturiello, Alessia Chiara Latini, Giulio Falasconi, Cesare de Gregorio, Pasquale Crea, Giuseppe Dattilo, Antonio Berruezo, Antonio Micari, Gianluca Di Bella

**Affiliations:** 1 Department of Clinical and Experimental Medicine, University of Messina, 98100 Messina, Italy; 2 Heart Institute, Teknon Medical Centre, 08022 Barcelona, Spain; 3 Cardiovascular Department, Clinical and Interventional Arrhythmology, ARNAS Ospedale Civico Di Cristina Benfratelli, 90127 Palermo, Italy; 4 Health Science Interdisciplinary Center, Scuola Superiore Sant’Anna, 56127 Pisa, Italy; 5 Department of Cardio-Thoraco-Vascular Care, Azienda Socio-Sanitaria Territoriale Lecco-Ospedale A. Manzoni, 23900 Lecco, Italy; 6 Humanitas Research Hospital IRCCS, 20089 Rozzano, Milan, Italy; 7 Department of Biomedical Sciences, Humanitas University, 20072 Pieve Emanuele, Milan, Italy; 8 Department of Biomedical and Dental Sciences and Morphological and Functional Imaging, University of Messina, 98100 Messina, Italy

**Keywords:** cardiomyopathy, hypertrophic cardiomyopathies, HCM, phenocopies, classification, diagnosis, differential diagnosis, sarcomeric cardiomyopathy, amyloidosis, Fabry disease

## Abstract

Among cardiomyopathies, the hypertrophic phenotype is the most common, and hypertrophic cardiomyopathy (HCM) phenocopies represent a heterogeneous group of conditions. They are defined by a left ventricular wall thickness ≥15 mm in the absence of other causes such as loading conditions, ischemia, or valvular disease. Although they mimic similar clinical and morphological features, their etiologies are distinct and include genetic, metabolic, and infiltrative mechanisms. Therefore, accurate classification and differential diagnosis are crucial for effective management and treatment. Sarcomeric HCM is the most frequent form, accounting for up to 60% of cases. However, numerous non-sarcomeric phenocopies exist, including amyloidosis, Fabry disease, glycogen storage disorders, RASopathies, and mitochondrial diseases. Clinical and imaging findings are essential to distinguish these entities from sarcomeric HCM. Electrocardiography, echocardiography, advanced modalities such as cardiac magnetic resonance (CMR), and specific laboratory tests all play a central role in guiding diagnosis. Genetic testing provides key insights into mutations and inheritance patterns, further supporting definitive diagnosis. Correct identification of an HCM phenocopy carries important therapeutic implications, as disease-specific treatments can significantly improve prognosis. For example, targeted therapies exist for amyloidosis, Fabry disease, and certain metabolic or mitochondrial disorders, underlining the clinical relevance of an accurate diagnosis. This review aims to provide an overview of HCM phenocopies and assist clinicians in diagnostic reasoning. The first part addresses classification according to pathophysiological mechanisms, clinical features, and genetic background. The second part focuses on the stepwise approach to differential diagnosis, integrating clinical assessment, laboratory evaluation, ECG, echocardiography, and CMR findings.

## 1. Introduction

**Figure 1:**
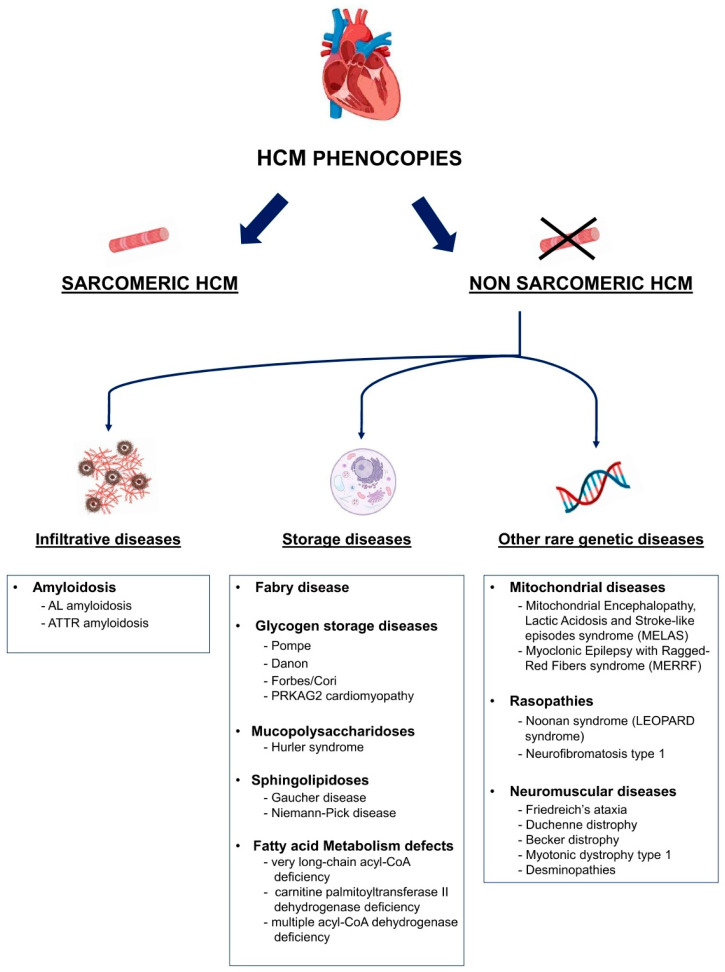
Proposed classification of main HCM phenocopies. AL: light-chain amyloidosis; ATTR: transthyretin amyloidosis; HCM: hypertrophic cardiomyopathy.

Cardiomyopathies are a heterogeneous group of myocardial diseases. They are traditionally classified according to phenotype as hypertrophic cardiomyopathy (HCM), dilative cardiomyopathies, and restrictive cardiomyopathies [1]. As highlighted by MOGE classification, the same phenotype can be caused by different etiologies and genetics, with different syndromic manifestations [2]. Therefore, differential diagnosis of cardiomyopathy phenocopies is crucial for correct disease management. This issue arises especially for HCM phenocopies, as they are numerous (Figure 1), and in this article we will focus on the key features within the current science with an original approach focusing on their diagnosis. First, HCM phenocopies will be presented in detail to provide an overview of the classification of each disease and its main characteristics. Second (in paragraph 10), we will adopt the opposite perspective, starting from the main disease findings (clinical, ECG, and echocardiographic) to offer the reader a quick practical guide for reaching a diagnosis in everyday clinical practice. Although current guidelines remain an essential reference and address cardiomyopathies in a comprehensive manner, from diagnosis to therapy, a dedicated theoretical and practical focus on the differential diagnosis of HCM phenocopies offers an extremely helpful guide for clinical decision-making, particularly in such a broad field [3].

**Definition and classification**

Hypertrophic phenotype is defined as a left ventricular wall thickness of 15 mm or more in at least one myocardial segment, measured by an imaging technique and not fully explained by loading conditions (hypertension, athlete’s heart), myocardial ischemia, or valve dysfunction (e.g., severe aortic stenosis) [3,4].

**Figure 2:**
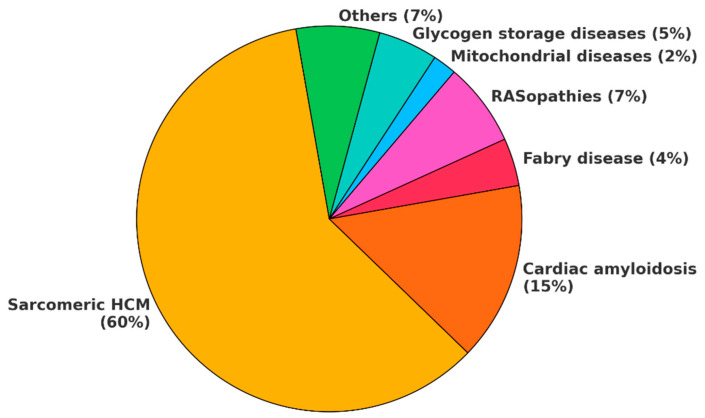
HCM phenocopy incidence. This chart illustrates the incidence of HCM phenocopies as currently reported in the literature. However, the incidence of cardiac amyloidosis is rapidly increasing due to improved disease awareness and enhanced ability to diagnose it at earlier stages. Some estimates suggest that, in the general population, cardiac amyloidosis may even be more common than sarcomeric HCM. In the chart, *others* include mucopolysaccharidoses, sphingolipidoses, fatty acid metabolism defects, neuromuscolar diseases, X-linked and autosomal muscular dystrophies. HCM: hypertrophic cardiomyopathy.
(v) **Other inborn errors of metabolism**: a hypertrophic phenotype can also be associated with inborn errors of metabolism like mucopolysaccharidosis (Hurler syndrome), sphingolipidosis (Gaucher and Niemann–Pick disease) and fatty acid metabolism defects
(vi) **Mitochondrial diseases**: heterogeneous group of diseases caused by mutations of the mitochondrial genome leading to dysfunctional energy production. They have multisystemic manifestations involving the central nervous system, heart, and skeletal system.
(vii) **RASopathies**: include neurofibromatosis type 1, Noonan syndrome, Costello syndrome, cardio-facio-cutaneous syndrome, and Legius syndrome. These syndromes are caused by germline mutations in genes that alter Ras subfamily genes.
(viii) **Neuromuscular diseases**: among them are Friedreich’s ataxia, muscular dystrophies such as Duchenne and Becker dystrophy or myotonic dystrophy type 1 or in association with muscle weakness and contractures caused by mutations of the Four and Half LIM domain-1 (*FHL-1*) gene.

HCMs have a prevalence ranging from 1:500 (0.2%) to 1:200 (0.5%) in the general population and the sarcomeric variant represents up to 60% of cases, while amyloidosis is the second most common etiology (Figure 2) [5,6,7]. The majority of HCM phenocopies are inherited conditions, as most of them arise from underlying genetic mutations [3].

Hypertrophic phenotypes include sarcomeric and non-sarcomeric cardiomyopathies. These latter can be further divided into infiltrative diseases, such as amyloidosis, storage diseases, such as Fabry disease, glycogen storage diseases, and mitochondrial diseases [1].

Following the most recent ESC classification, the HCM phenocopies can be classified as follows (Figure 1) [3,7,8,9,10]:

(i) **Sarcomeric HCM**: an inherited form of HCM caused by mutations in genes encoding sarcomeric proteins of the cardiac muscle.
(ii) **Cardiac amyloidosis**: due to extracellular amyloid deposition, causing an infiltrative HCM with a restrictive evolution in the more advanced stages. The two most common forms are light chain amyloidosis (AL) and transthyretin amyloidosis (ATTR).
(iii) **Anderson–Fabry disease**: a lysosomal storage disorder due to a mutation in the α-galactosidase A gene with accumulation of glycosphingolipids on cardiomyocytes. It is an X-linked recessive disease and can occur with cardiological, renal, neurological (peripheral and central), and dermatological manifestations.
(iv) **Glycogen Storage Disorders (GSD)**: characterized by hypertrophy due to glycogen-filled vacuoles in the cells. Among them, they include Danon disease, Pompe disease (GSD type II), Forbes disease (GSD type III), and PRKAG2 cardiomyopathy. In these cases, multisystemic clinical features at an early age are often observed, together with an extreme left ventricular hypertrophy and progression to dilated cardiomyopathy and electrocardiographic abnormalities, such as ventricular pre-excitation and conduction system defects.

**Figure 3:**
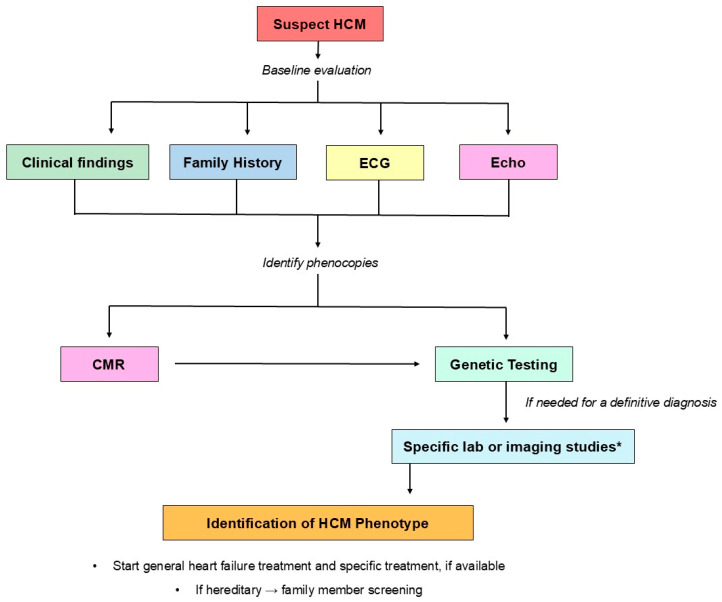
Proposed HCM phenocopy overall diagnostic algorithm. The diagnostic algorithm for HCM phenocopies can be divided into two parts. First, when HCM is suspected, clinical findings, family history, ECG, and echocardiography provide a baseline assessment to confirm the HCM phenotype and to raise suspicion of a specific phenocopy. Second, CMR and genetic testing are needed to perform a non-invasive first-line differential diagnosis among phenocopies. Depending on the specific HCM phenocopy, additional laboratory tests or imaging studies may be required to establish a definitive diagnosis. * For a definitive diagnosis of certain HCM phenocopies, specific blood tests or imaging studies are required. For example, the diagnosis of transthyretin cardiac amyloidosis (ATTR) requires serum and urine protein electrophoresis with immunofixation and free light chain assessment to exclude light chain amyloidosis (AL) and bone scintigraphy for a definitive diagnosis. CMR: cardiac magnetic resonance; ECG: electrocardiogram; Echo: echocardiography; HCM: hypertrophic cardiomyopathy.

Diagnosis suspicion should be guided by familial history and clinical red flags which are different for each hypertrophic phenocopy (Figure 3). However, absence of signs and symptoms does not exclude the diagnosis and unfortunately sudden cardiac death (SCD) can be the first HCM manifestation. Electrocardiogram (ECG) can support HCM suspicion and rhythm alterations are frequent, especially atrial fibrillation.

Echocardiography is the first-level imaging exam for HCM diagnosis, showing left ventricle hypertrophy and diastolic dysfunction as main features. Other common findings are left atrium enlargement, systolic disfunction evaluated by global longitudinal strain (GLS), and left ventricle outflow tract (LVOT) obstruction due to systolic anterior motion (SAM) of the mitral valve [1,4]. Once a hypertrophic heart is observed by echocardiography, cardiac magnetic resonance (CMR) can confirm morphological characteristics and is an ideal tool for differential diagnosis of HCM phenocopies, thanks to multiparametric tissue characterization [11]. Finally, genetic testing and specific lab or imaging studies, such as bone scintigraphy for ATTR, are needed for some definitive diagnosis of when a specific HCM phenocopy is suspected because of previous evaluations (Figure 3).

## 2. Sarcomeric HCM

**Figure 4:**
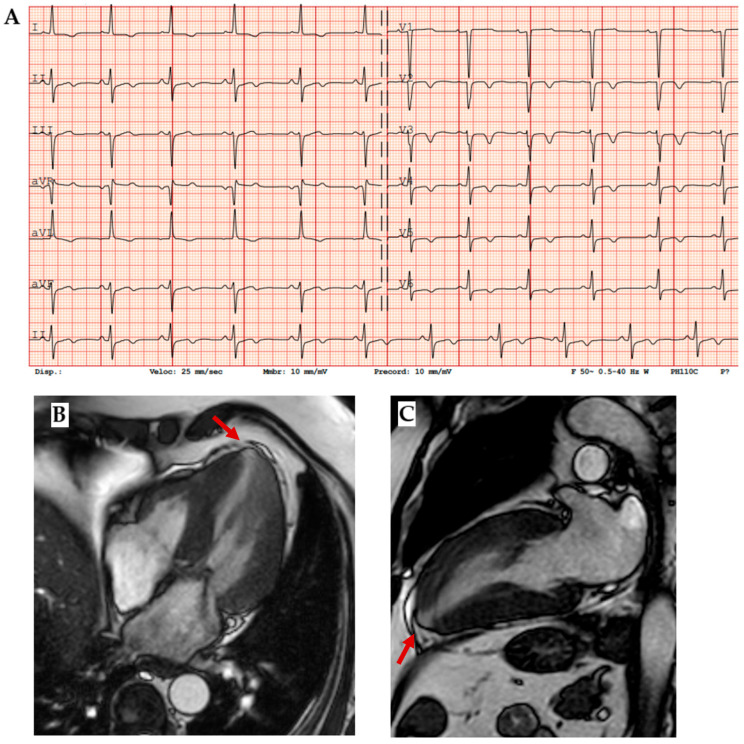
Sarcomeric HCM. (**A**) ECG showing diffuse negative T waves, especially in precordial leads. (**B**) Mid-ventricular short axis cine-bSSFP image and (**C**) 2-chamber cine-bSSFP image of a patient with *MYH 7* mutation (*p.Arg403Gln*), cardiac hypertrophy, and apical aneurism, which is a typical finding in sarcomeric HCM (red arrows).

Sarcomeric HCM (most common OMIM 192600) covers up to 60% of HCM phenocopies in adolescents and adults. It has an autosomal dominant inheritance with highly variable clinical expression ranging from asymptomatic forms to heart failure or SCD. Up to 70% of sarcomeric HCMs are caused by known mutations in cardiac sarcomere and sarcomere-related protein genes with an autosomal dominant inheritance. Myosin-binding protein C (*MYBPC3*) and beta-myosin heavy chain (*MYH7*) (Figure 4A) are the most common genes involved. Mutations in these two genes together with mutations in the cardiac troponin T (*TNNT2*), cardiac troponin I (*TNNI3*), and tropomyosin alpha-1 chain (*TPM1*) genes have been observed in around 60% of patients [3,12,13]. Other genes involved in sarcomeric HCM are *ACTC1* (cardiac α-actin), *MYL2* (myosin light chain 2), *MYL3* (myosin light chain 3), and *CSRP3* (Cysteine and Glycine Rich Protein 3). Penetrance and expression are variable. Therefore, the age of onset varies widely from childhood to adulthood, the clinical course of sarcomeric HCM is difficult to predict, and disease risks related to single mutations are under investigation [3,14,15].

Symptoms related to sarcomeric HCM can be classified as related to heart failure (exertional dyspnea or fatigue), microvascular ischemia (chest pain), and arrhythmias (palpitations). LVOT obstruction, syncope, pre-syncopal episodes, and familial history for previous symptoms or for SCD are important red flags.

ECG is a very sensitive tool which is abnormal in 90% of cases (Figure 4A). Typical findings are high-voltage QRS and deep Q waves as expression of cardiac hypertrophy and pseudonecrosis, respectively; diffuse repolarization abnormalities as giant negative T waves, supraventricular (ex: preexcitation), and ventricular arrhythmias are also common. In echocardiography, beyond different patterns of cardiac hypertrophy (septal, apical, and diffuse), diastolic dysfunction, mitral regurgitation, left ventricle obstruction, systolic anterior motion of mitral valve (SAM), and abnormal longitudinal strain in hypertrophic segments are typical findings [16,17]. High-resolution CMR allows for detection of apical aneurysms (Figure 4B,C) and myocardial crypts; late gadolinium enhancement (LGE) shows mid-wall enhancement in hypertrophic segments and T1 and ECV are increased in fibrotic areas [11]. In biopsy, disarray of hypertrophied myocytes and interstitial fibrosis are the main histologic characteristics.

When clinical or instrumental suspicion of sarcomeric HCM arise, genetic testing is the gold standard for a definitive etiological diagnosis. Furthermore, a genetic test can be useful in relatives of sarcomeric HCM patients, especially when red flags are present.

Sarcomeric HCM is associated with SCD in 1% of patients because of malignant ventricular arrhythmias or severe LVOT obstruction. Therefore, it results in a leading cause of genetic and heritable heart disease and SCD in young people [3,12,18]. Once HCM is diagnosed, a multiparametric SCD risk score validated by the European Society of Cardiology (ESC) can be calculated to guide patients’ follow-up and management. Heart failure progression and arrhythmia management (especially atrial fibrillation) are also crucial. Furthermore, mavacamten, a myosin modulator, has recently joined traditional surgical and medical tools for obstructive HCM management [19].

## 3. Amyloidosis

Amyloidoses are a group of progressive infiltrative diseases characterized by the extracellular deposition of mis-folded proteins in many possible organs including the heart, kidneys, and peripheral neurons. It was previously considered a rare disease, with an incidence of 30 cases per million per year, accounting for 10–15% of HCM cases. However, in recent years, greater awareness of the disease and the standardization of diagnostic methods have led to a significant increase in diagnoses [20]. It occurs in 10% of heart failure cases and some estimates even suggest that, when including subclinical forms, it may affect up to 1% of the general population, especially elderly individuals, making it the most common form of HCM [21].

Even if more than 30 amyloidogenic proteins have been described, myocardial deposits of amyloid fibrils have been observed in vivo only for nine precursor proteins. More than 98% of newly diagnosed cardiac amyloidosis is due to the deposition of fibrils composed of monoclonal immunoglobulin light chains (AL, OMIM 105200) or transthyretin (ATTR, OMIM 105210), either wild-type (ATTRwt) or mutated (ATTRm) [22]. Therefore, AL usually occurs in the context of hematological diseases as multiple myeloma, monoclonal gammopathy of undetermined significance (MGUS), or Waldenström’s macroglobulinemia. On the other hand, transthyretin is a protein involved in T4 and vitamin A transport through the bloodstream and its accumulation is due to defects in its production (therefore in its three-dimensional structure) because of advanced age or an inherited mutation. Regardless of the underlying pathogenesis of amyloid production, cardiac involvement remains the leading cause of morbidity and mortality, underscoring the need for early diagnosis and treatment [23]. Infiltration can also affect both vessels, leading to reductions in myocardial perfusion, and the conduction system, causing atrioventricular or intraventricular conduction delays [24,25,26].

Age at diagnosis or first presentation may be useful for distinguishing the etiology of amyloidosis: AL is typical of the sixth or seventh decade, ATTRwt is typically a disease of elderly people especially from the eighth decade, while ATTRv can occur from the third decade, most commonly after the age of 40 years. Sensory abnormalities such as dysesthesia, typically in a glove and stocking distribution, and neuropathic pain are common both in AL and ATTR, while the association between HCM phenotype and bilateral carpal tunnel syndrome is more suggestive of transthyretin-related amyloidosis, which may precede cardiac symptoms by 10–15 years [7]. Electrocardiographic evaluation may be suspicious towards cardiac amyloidosis. Indeed, the presence of low QRS voltage or normal voltage despite cardiac hypertrophy and a pseudo-infarction pattern, despite normal coronary arteries, are typical findings. Furthermore, conduction system abnormalities, as well as atrial and ventricular arrhythmias (especially atrial fibrillation), are common [26].

**Figure 5:**
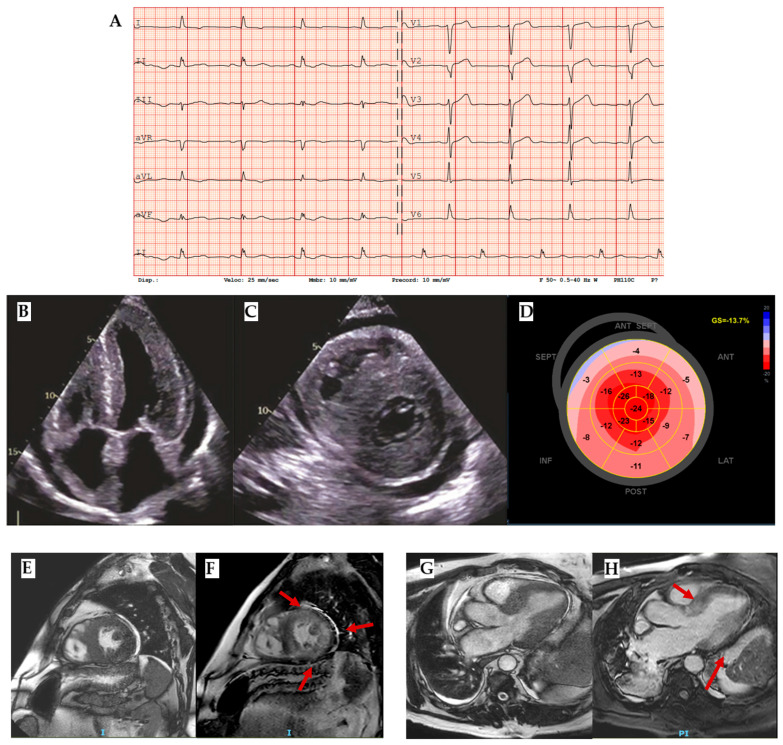
Amyloidosis. (**A**) ECG with an important mismatch between cardiac hypertrophy and electrical voltage in a patient with ATTR and severe cardiac hypertrophy with 23 mm septum thickness: low voltages are observed in peripheral leads and normal voltages in precocardial leads. (**B**) Four chamber and (**C**) short-axis 2 chamber showing not only left ventricle hypertrophy but also other typical findings in amyloidosis such as right ventricle hypertrophy, thick interatrial septum, Coumadin ridge, and cardiac valves. (**D**) Reduced Global Longitudinal Strain (GLS) with apical sparing and the typical cherry-on-the-top sign. (**E**) Cine-bSSFP short-axis 2-chamber view, (**F**) LGE short-axis 2-chamber view (red arrows highlight the LGE segments), (**G**) Cine-bSSFP 3-chamber view, and (**H**) LGE 3-chamber view of a patient with severe cardiac ATTR with transmural LGE in all mid segments except the inferior septum and basal antero-septum and infero-lateral walls (red arrows). ATTR: transthyretin amyloidosis.

Amyloidosis involves the heart as a whole. Indeed, on an echocardiogram, it is characterized by an interventricular septum diameter greater than 12 cm, a granular sparkling appearance, normal to small LV cavity size (restrictive physiology), biatrial enlargement and dysfunction with left atrial and left appendage stasis and thrombi, interatrial septal and right ventricular thickening, pericardial effusion, diastolic dysfunction with restrictive transmitral Doppler filling pattern (steep deceleration time, low tissue Doppler velocity at the mitral annulus and an elevated E/e’ ratio), and aortic stenosis [27] (Figure 5B–D).

Even in the earlier phases of the disease there is impairment of longitudinal systolic function, while radial thickening and circumferential shortening are still preserved. This feature can be analyzed by speckle tracking echocardiography, with the evidence of abnormal longitudinal strain in the basal and mid segments with relative preservation in the apical segments, as shown in the Global Longitudinal Strain bullseye map with the cherry-on-the-top sign (Figure 5D) [23,28].

Tissue characterization provided by CMR is fundamental in the diagnostic work-up of amyloidosis, helping in differential diagnosis. The main features are high native T1 and LGE with a subendocardial or a transmural pattern (Figure 5E–H) [11]. Radionuclide imaging, especially bone scintigraphy with the use of 99mTc-labeled diphosphonate, plays a central role in the noninvasive diagnosis of ATTR cardiac amyloidosis, with high sensitivity and specificity, especially for Perugini’s score Grade 2–3 (heart capitation of the tracer visually equal or super than that in bone). Indeed, according to the consensus algorithm proposed by Gillmore et al. for the differential diagnosis between AL and ATTR, serum/urine immunofixation, serum light chain assay, and bone scintigraphy are the mandatory steps for noninvasive diagnosis [29]. On the other hand, the role of nuclear imaging, particularly PET, in the diagnosis of cardiac amyloidosis and its potential for distinguishing between AL and ATTR is under investigation [30].

Cardiac amyloidosis has a great impact on patient quality of life [31,32]. For AL, treatment consists of managing the hematologic disease with targeted chemotherapy, while for ATTR, the treatment involves prevention of cardiac complications (heart failure, arrhythmias, conduction disturbances, thromboembolism, and concomitant presence of severe aortic stenosis) and disease-modifying therapies to further stop amyloid deposition. Among them, the main drugs currently on the market are stabilizers of circulating transthyretins (tafamidis) and genetic silencers (patisiran, inotersen, and acoramidis) [33]. Tafamidis is generally the agent of choice in ATTR cardiac patients with reasonable expected survival, while patisiran is usually considered in ATTRm patients with both cardiac and neurological involvement [34].

## 4. Fabry Disease

Fabry disease (OMIM 301500), also known as Anderson–Fabry disease, is an X-linked lysosomal storage disorder caused by mutations in the α-galactosidase A (GLA) gene. These mutations result in reduced or absent activity of the α-galactosidase A (AGAL-A) enzyme, leading to progressive accumulation of glycosphingolipids—primarily globotriaosylceramide (Gb3) and its deacylated form, globotriaosylsphingosine (lyso-Gb3)—within various cell types, including vascular endothelial cells, smooth muscle cells, and cardiomyocytes. Absent or reduced enzyme activity leads to the inability to catabolize globotriaosylceramide (Gb3) and related glycosphingolipids, with the result of a progressive intracellular storage of Gb3 in various tissues and organs and an elevated plasma concentration of lyso-Gb3. The most commonly affected organs are the heart, vascular endothelium of the kidney, nervous system, eyes, and skin [35].

**Figure 6:**
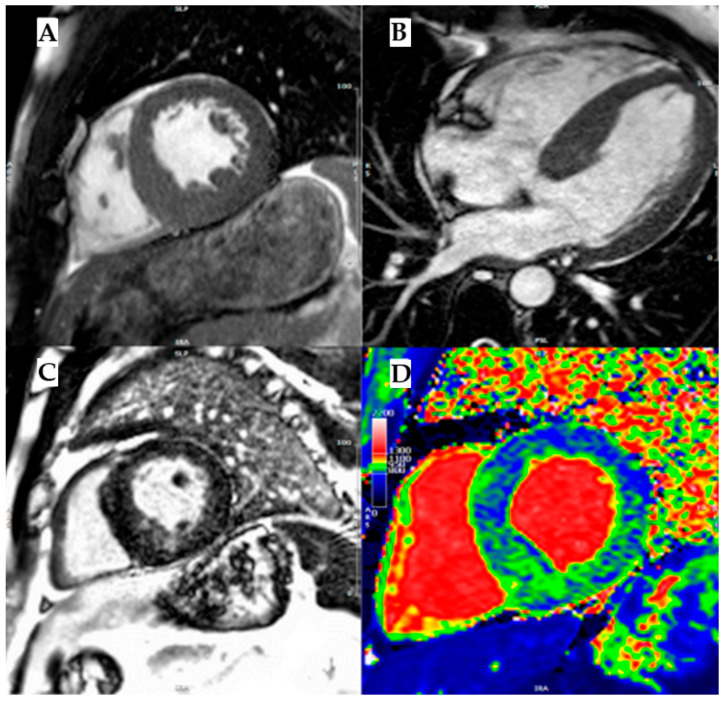
Fabry disease. (**A**) Mid-ventricular short axis cine-bSSFP image in telediastole and (**B**) cine-bSSFP 4- chamber image in telediastole, both showing a case of Anderson–Fabry disease with asymmetric septal LV hypertrophy. (**C**) Mid-ventricular short axis late gadolinium enhancement (LGE) image showing mid-wall LGE in inferoseptum and inferior wall. (**D**) Mid-ventricular short axis T1 map showing a diffuse reduction in intra-myocardial T1 values, with T1 pseudo-normalization in segments with LGE. Reproduced with permission from *Licordari, R. J*. *Clin*. *Med. 2023* [11].

As it is an X-linked disease, men are more commonly affected than women, who show a mild disease phenotype in heterozygosis or because of lyonization. The disease incidence is estimated at one case per 50,000–100,000 people. Early signs and symptoms usually manifest during childhood and adolescence in patients with the severe form of Fabry disease (men or women in homozygosis) and may include neuropathic pain with a disto-proximal progression, autonomic dysfunction, gastro-intestinal complaints, angiokeratomas, and hypohidrosis. These precede the development of kidney dysfunction and cardiac and cerebrovascular complications in adulthood, which cause poor quality of life and an increased risk of premature death. However, clinical red flags are often recognized late, and diagnosis is not uncommon in patients with advanced disease manifestations such as kidney insufficiency under dialysis treatment, heart failure, or ischemic ictus. Cardiac manifestations are common in Fabry disease, occurring in 40–60% of patients. The classic cardiac involvement is a concentric left ventricular hypertrophy, but it can also present as asymmetrical septal or obstructive. The age of onset for full cardiac involvement is strictly dependent on the level of residual AGAL-A activity related to the genetic variant. In men, hypertrophy develops usually after the third or fourth decade of life, while in women the onset of cardiomyopathy is delayed by 10 years [36]. CMR typically shows LGE in the basal inferolateral wall and low native T1 caused by fat deposits (Figure 6).

Although the mechanism underlying this regional pattern of LGE remains uncertain, the presence of low native T1 is particularly important, as it is specific for fat infiltration, the main pathognomonic feature of Fabry disease, which is characterized by lipid deposition.

As for disease screening, enzymatic activity tests are good tools in men, while in women lyso-Gb3 dosage should be preferred because of possible residual enzyme activity. For definitive diagnosis genetic testing is needed.

Enzyme replacement therapy is the traditional specific treatment for Fabry disease. However, since 2016, the pharmacological chaperone migalastat has also been available in Europe. In selected mutations, it promotes proper folding of the AGAL-A enzyme, restoring its activity. Both treatments can reverse disease progression in the early stages and should therefore be initiated as soon as possible. However, their effectiveness is limited in advanced disease, when irreversible organ damage has already occurred. Even if enzyme replacement therapy is effective, regardless of the causative mutation, it is administered as an intravenous infusion each two weeks, while migalastat is taken orally every other day [37].

## 5. Glycogen Storage Diseases

Glycogen storage diseases (GSDs) encompass a heterogenous group of metabolic disorders caused by pathogenic variants in genes encoding enzymes involved in glycogenesis, glycogenolysis, or glycolysis [38]. In these disorders, genetic mutations result in excessive glycogen accumulation in multiple organs, primarily affecting the liver, skeletal muscles, and myocardium. Among GSDs, Pompe disease (GSD type IIa), Danon disease (GSD type IIb), Cori disease (GSD type III), and PRKAG2 disease are particularly notable for their association with HCM that manifests during childhood [39]. Pompe disease should be considered in the differential diagnosis of infants exhibiting multiorgan involvement, highlighted by severe biventricular hypertrophy, hepatomegaly, elevated serum transaminases and creatine kinase levels, hypotonia, and delayed motor development [40]. In contrast, both Danon disease and PRKAG2 disease usually emerge after the first year of life, with Danon disease exhibiting an earlier onset in male patients and presenting with muscle involvement and cognitive impairments [41].

**Glycogen storage disease type IIa: Pompe disease**

Pompe disease (OMIM 232300), or glycogen storage disease type II (GSDII), is an autosomal recessive lysosomal storage disorder caused by mutations in the *GAA* gene encoding acid alpha-glucosidase. This enzyme is responsible for degrading glycogen into glucose within the lysosomes. Its deficiency leads to pathological glycogen accumulation in various tissues, particularly in cardiac, skeletal, and smooth muscle with an infantile-onset or a late-onset form related to the degree of residual enzyme activity. Although classically considered a metabolic myopathy, Pompe disease may present as a phenocopy of HCM, especially in its infantile-onset form, where massive biventricular hypertrophy is a hallmark finding.

The infantile-onset form typically manifests within the first few months of life with profound hypotonia, feeding difficulties, macroglossia, respiratory insufficiency, and rapidly progressive hypertrophic cardiomyopathy. The cardiac phenotype is characterized by concentric left ventricular hypertrophy, often with involvement of the right ventricle, and may mimic sarcomeric HCM on echocardiography. Electrocardiographic findings frequently show short PR interval, tall QRS complexes, and ventricular pre-excitation, which are suggestive but not pathognomonic. Without enzyme replacement therapy (ERT), the disease progresses rapidly to heart failure and death within the first year of life.

In contrast, the late-onset form (LOPD) presents from childhood to adulthood with a more indolent course and predominant skeletal muscle involvement, though mild cardiac hypertrophy or electrical abnormalities (e.g., conduction delays and arrhythmias) can still occur. As such, it may be overlooked in the differential diagnosis of unexplained muscle weakness or mild HCM, especially when the classic infantile features are absent.

Diagnosis is established by reduced GAA enzymatic activity in dried blood spots, leukocytes, or fibroblasts, and confirmed by genetic testing of the *GAA* gene. Ancillary findings supporting the diagnosis include elevated creatine kinase, abnormal liver enzymes, and, in infantile forms, echocardiographic features of HCM. Cardiac MRI can help identify increased wall thickness and may reveal characteristic findings such as diffuse or patchy LGE in advanced stages, although imaging alone cannot differentiate Pompe disease from other infiltrative or storage cardiomyopathies.

Enzyme replacement therapy (ERT) with recombinant human acid alpha-glucosidase has revolutionized the management of Pompe disease. When initiated early, especially in infantile-onset patients, ERT can significantly improve cardiac structure and function, delay disease progression, and enhance survival. In late-onset forms, ERT slows the decline of motor and respiratory function, although its impact on cardiac outcomes is generally less pronounced. Given the potential reversibility of cardiac involvement with early treatment, Pompe disease should be systematically considered in the differential diagnosis of HCM in infants, particularly when associated with hypotonia or systemic signs of metabolic disease. Awareness of this phenocopy is crucial, as early detection allows for life-saving interventions and appropriate genetic counseling [42,43,44].

**Glycogen storage disease type IIb: Danon disease**

Danon disease is a rare X-linked dominant disorder which represents up to 2% of all HCM and has to be considered especially in young patients with massive left ventricular hypertrophy and conduction abnormalities. Even if it was traditionally classified as a glycogen storage disease, it is not caused by a primary disorder of glycogen metabolism, it is caused by mutations in the *LAMP2* gene, which encodes lysosome-associated membrane protein 2, essential for normal autophagy and lysosomal function. Dysfunction of this pathway leads to intracellular accumulation of autophagic vacuoles, especially in cardiac and skeletal muscle, and often in neurons, resulting in a multisystemic phenotype. Therefore, it has recently been proposed that it should not be included among the glycogen storage disorders. The disease typically presents in adolescent males with a triad of severe cardiomyopathy, skeletal myopathy, and intellectual disability, while female carriers often display a more isolated cardiac involvement with a later onset and milder course. While it accounts for less than 1% of cases classified as HCM, Danon disease is of relevance due to its severe clinical course, systemic involvement, and implications for patient management and family screening.

The cardiac manifestations of Danon disease are often indistinguishable from sarcomeric HCM on initial clinical evaluation, with left ventricular wall thickness frequently exceeding that seen in typical HCM cases. However, several red flags can guide the suspicion: early onset (often in the second decade), rapid progression to heart failure, conduction system disease (including pre-excitation), atrial arrhythmias, and an unusually high incidence of SCD. Electrocardiographic findings frequently include Wolff–Parkinson–White (WPW) patterns, high voltages, and repolarization abnormalities. On imaging, echocardiography and cardiac MRI typically show marked concentric or asymmetric hypertrophy, small ventricular cavities, diastolic dysfunction, and often extensive LGE, reflecting myocardial fibrosis. The imaging phenotype can overlap with other infiltrative or storage diseases such as Fabry disease or cardiac amyloidosis, making differential diagnosis essential.

Genetic testing remains the gold standard for diagnosis. Muscle biopsy may show vacuole myopathy with glycogen accumulation and absence of *LAMP2* expression on immunohistochemistry, although these findings are less commonly used today due to the availability of molecular diagnostics. Elevated serum creatine kinase and hepatic transaminases are supportive laboratory findings.

From a clinical standpoint, the course is aggressive, especially in males, with many patients progressing to end-stage heart failure requiring cardiac transplantation in their twenties. There is no specific therapy currently available targeting the underlying pathophysiology, and management is largely supportive, including standard heart failure therapy, arrhythmia surveillance, and consideration for implantable cardioverter—defibrillators (ICDs) and transplantation. Given its severity and genetic implications, Danon disease should always be considered in the differential diagnosis of early-onset, rapidly progressive HCM-particularly when associated with neuromuscular symptoms, cognitive impairment, or ECG evidence of pre-excitation [45,46,47].

**Glycogen storage disease type III: Forbes disease or Cori disease**

Forbes disease (OMIM 232400), also referred to as glycogen storage disease type III (GSD III), is a rare autosomal recessive metabolic disorder. It is characterized by a deficiency in glycogen debranching enzyme (GDE) activity, resulting in the accumulation of abnormal glycogen. The estimated prevalence is approximately 1 in 100,000 live births [48]. GSD III arises from mutations in the AGL gene located on chromosome 1p21, which leads to impaired GDE function responsible for glycogen breakdown. This enzyme deficiency can affect both the liver and muscle (GSD IIIa) or be confined to the liver alone (GSD IIIb).

The clinical manifestations typically appear in early childhood, results from impaired glycogen degradation and excessive glycogen accumulation. These include liver dysfunction, hypoglycemia, significant hepatomegaly, cirrhosis, and hypoglycemia-induced seizures. Additionally, skeletal muscle myopathy, hypertrophic cardiomyopathy, and impaired growth are commonly observed in patients with form IIIa. Cardiac involvement in GSD IIIa varies among individuals and even if ventricular hypertrophy is commonly observed (30% to 80% of cases) significant cardiac dysfunction is rare. However, sudden death, likely due to cardiac arrhythmias, has been reported.

Typical biochemical abnormalities observed in Forbes disease include low blood sugar levels without acidosis, high triglyceride levels, elevated liver enzyme levels, the presence of ketones, and increased creatine kinase levels. Diagnosis is confirmed through the identification of excessive and structurally irregular glycogen deposits characterized by shorter outer branches and reduced activity of the debranching enzyme in biopsy samples or blood cells. Endomyocardial biopsy typically reveals glycogen accumulation without myocyte disarray, differing from the histological features commonly seen in hypertrophic cardiomyopathy caused by sarcomeric mutations. Another diagnostic approach involves detecting pathogenic mutations in the AGL gene on both alleles. The most frequently considered alternative diagnosis in this context is GSD type Ia, which is attributed to a malfunction of the glucose-6-phosphatase enzyme [49,50].

Hyperlipidemia, encompassing high cholesterol and triglyceride levels, is prevalent among patients with GSD III. Consequently, there may be a risk of vascular dysfunction leading to early atherosclerosis or early coronary artery disease in these individuals, although data on this clinical issue are very limited and inconsistent [49].

While ECG findings may show signs of ventricular hypertrophy, specific rhythm disturbances are generally rare. Echocardiographic studies of individuals with GSD III have reported findings related to left ventricular hypertrophy with diastolic dysfunction as the first functional abnormality to arise. Serial echocardiograms should be recommended beginning at the time of diagnosis and repeated every 12–24 months for patients with GSD IIIa and every 5 years in patients with GSD IIIb [49].

From a prognostic and treatment perspective, predictive characteristics are not well understood, and no correlation has been found with myopathy or creatine kinase activity. There is not a specific therapy, and the management is based on a specific high-protein diet to facilitate gluconeogenesis, with enteral nasogastric or parenteral feeding at night in case of hypoglycemia, together with frequent meals and supplements. Caution should be used in prescribing beta-blockers due to their risk of hypoglycemia and statin which could worsen or trigger myopathy [50,51].

**PRKAG2 disease**

*PRKAG2* syndrome (OMIM 600858) is a rare, early-onset, autosomal dominant genetic disorder marked by ventricular pre-excitation, supraventricular arrhythmias, and cardiac hypertrophy. It is caused by pathogenic variants in the *PRKAG2* gene. This gene encodes the gamma-2 regulatory subunit of AMP-activated protein kinase, integral to proper glucose metabolism and glycogen storage within the myocardium. The prevalence is currently unknown, but it may be rising because of the growing availability of genetic testing for HCM.

In 2001, Gollob et al. identified the causative mutations responsible for this syndrome in the *PRKAG2* gene, which encodes the γ2 regulatory subunit of 5′ AMP-activated protein kinase (AMPK), a key enzyme in cellular energy regulation [52].

Affected individuals often experience chronotropic incompetence and advanced heart block, frequently requiring early pacemaker implantation. Cardiac hypertrophy, which is typically symmetrical and involves the left ventricle, can be significant but is rarely linked to left ventricular outflow tract obstruction.

**Figure 7:**
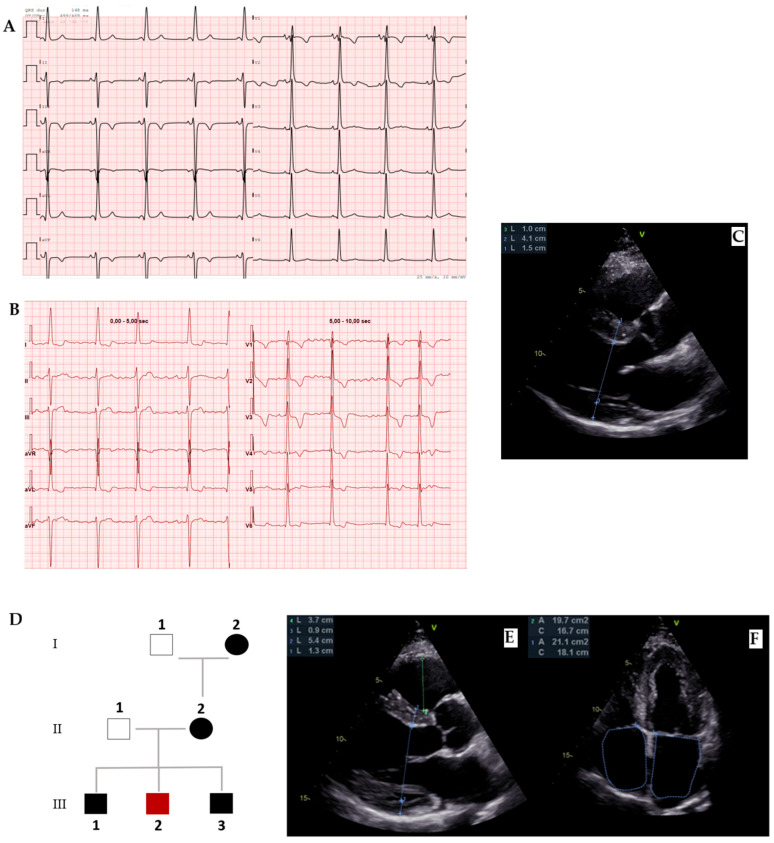
*PRKAG2* disease. (**A**) Forty-one-year-old male diagnosed with *PRKAG2* syndrome because of ventricular pre-excitation, (**B**) paroxysmal atrial fibrillation, and (**C**) cardiac hypertrophy in young age as red flags. (**D**) The definitive diagnosis was confirmed by genetic testing (*p.Arg302Gln* mutation). Family screening with ECG and echocardiography was initiated from the proband (II-2) and led to early diagnosis in two brothers, as well as in the mother and maternal grandmother, demonstrating an autosomal dominant transmission of the mutation. (**E**,**F**) Echocardiogram of III-3, a 36-year-old male showing early cardiac hypertrophy.

The clinical presentation of *PRKAG2* syndrome can vary widely, with some patients displaying mild ventricular hypertrophy and arrhythmias, while others may be asymptomatic or at risk of SCD. In some cases, the syndrome may progress to heart failure (HF) or involve other systemic manifestations. The most frequently observed electrocardiographic finding is ventricular pre-excitation (Figure 7A), which appears in over half of the patients, often accompanied by bundle branch block, particularly affecting the right bundle. This seems to be related to both glycogen-induced structural remodeling of the AV junction and conduction system, favoring the development and unmasking accessory pathways. Additionally, slurred QRS complexes and atypical patterns of intraventricular conduction delays have been noted. Advanced atrioventricular or sinoatrial blocks are also commonly seen, as well as early-onset atrial fibrillation. High-voltage QRS complexes with secondary repolarization abnormalities frequently occur, even in the absence of echocardiographic left ventricular hypertrophy (Figure 7). Even if the most frequent arrhythmic problems are supraventricular arrythmias, characteristically this syndrome leads to advanced AV blocks, marked sinus bradycardia, or sinus blocks, even within the third or fourth decade of age: these conditions lead to pacemaker implantation in more than 40% of patients observed. Cardiac hypertrophy predominantly affects the left ventricle, characterized by a symmetrical and progressive nature, often accompanied by both diastolic and systolic dysfunction (Figure 7) [53]. Although the maximum ventricular wall thickness can vary and may be significant, it is infrequently associated with left ventricular outflow tract obstruction [54]. However, a restrictive mitral inflow Doppler pattern, hemodynamically significant left ventricular outflow tract obstruction, and progressive dilation could be major contributors to the need for cardiac transplantation or SCD. Additionally, no specific patterns related to cardiovascular magnetic resonance imaging have been identified in association with this syndrome [54,55]. Extracardiac involvement is rare and could determine skeletal myopathy and arterial hypertension.

Differential diagnoses are HCM without sarcomeric mutations and especially genetic syndromes like Danon’s disease and Fabry disease. Even if not cleared from a pathophysiologic point of view, SCD occurred in about 9% of patients observed, with a mean age of death of about 33 years, and can occur both in the presence and the absence of severe cardiac hypertrophy.

In the management of patients with this condition, it is important to highlight that symptom onset typically occurs within the first three decades of life and that specific treatment guidelines are lacking. Ultrasound imaging and cardiovascular magnetic resonance are the most effective techniques for identifying and characterizing cardiac hypertrophy. Antiarrhythmic therapy should be initiated for patients experiencing supraventricular or ventricular tachyarrhythmias. Pacemaker implantation should be considered when appropriate [55,56]. Although implantable cardioverter-defibrillator (ICD) placement may be an option, there is insufficient data regarding optimal patient selection. Heart transplantation may be indicated for patients with end-stage heart failure. It is essential to remember that genetic testing is necessary for both diagnosing the condition and screening family members when appropriate [55,56].

## 6. Other Inborn Metabolism Errors

Other rare metabolic disorders can be related to HCM. Among them, mucopolysaccharidoses, sphingolipidoses, and fatty acid metabolism defects are the most representative. These conditions are often multisystemic and may lead to cardiac hypertrophy through storage accumulation or impaired myocardial energy utilization.

**Mucopolysaccharidoses**

Mucopolysaccharidoses (MPS) are a group of lysosomal storage diseases caused by genetic defects in enzymes responsible for the degradation of glycosaminoglycans (GAGs). The absence or dysfunction of these enzymes leads to the progressive accumulation of GAGs within lysosomes, resulting in cell damage and multi-organ dysfunction: skeletal (dysostosis multiplex, short stature), neurological (cognitive impairment, hydrocephalus), cardiac (storage cardiomyopathy, valvular disease), respiratory (upper airway obstruction, sleep apnoea), and ophthalmologic (corneal clouding). These syndromes include

Hurler syndrome (MPS I, OMIM 252800), Hunter syndrome (MPS II, OMIM 309900), Sanfilippo syndrome (MPS III), and others. They are autosomal recessive disorders except for MPS II, which is X-linked and can be characterized by multiorgan involvement.

Between them, **Hurler syndrome** (MPS I) is the most common. It is a genetic disorder caused by a deficiency of the enzyme alpha-L-iduronidase, leading to the accumulation of glycosaminoglycans (GAGs) in the body. This accumulation results in progressive damage to various organs, causing developmental delays, skeletal deformities, respiratory problems, and heart issues. Cardiac valve thickening and dysfunction and hypertrophy are commonly present. Generally, left ventricle hypertrophy and consequent diastolic dysfunction appear at an early stage of the disease and have been described in up to 10% of patients with MPS [57]. It is important to highlight that left ventricle hypertrophy may also be a consequence of valvular disease. Also, conduction abnormalities, coronary artery abnormalities, and other vascular involvement such as systemic or pulmonary hypertension may occur. Systemic therapies, like hematopoietic stem cell transplantation or enzyme replacement therapy, may alter natural progression even with reduction in ventricular hypertrophy and recovery of ventricular function [58,59].

**Sphingolipidoses**

**Gaucher disease** (GD, OMIM 230800) is a lysosomal storage disorder caused by a deficiency of the enzyme glucocerebrosidase. This results in the accumulation of a fatty substance, the glucocerebroside, in cells and organs, particularly affecting the spleen, liver, and bone marrow. Symptoms vary depending on the type and can include enlarged organs, bone pain, anemia, and fatigue. The incidence of cardiac involvement in patients with Gaucher disease is considered uncommon but a reliable prediction is not available, and this consideration is almost entirely based on case reports. Cardiovascular manifestations might include pulmonary hypertension, calcification and thickening of valves and aorta, decreased cardiac output and left ventricle diastolic dysfunction due to myocardial infiltrative damage, restrictive cardiomyopathy, left ventricular hypertrophy, pericardial calcification and constrictive pericarditis. Predisposing factors, together with best therapeutic options, are not well understood [60,61].

**Niemann–Pick Disease** refers to a group of inherited disorders caused by the abnormal metabolism of lipids. In types A (OMIM 257200) and B (OMIM 607616), a deficiency of the enzyme acid sphingomyelinase leads to lipid accumulation in organs like the liver and spleen. Type C (OMIM 257220-607625) is caused by issues with lipid transport within cells. Symptoms can include neurological decline, difficulty moving, liver dysfunction, and respiratory problems. Cardiac involvement may include cardiomegaly with thickened LV walls and endocardial fibroelastosis, together with hypercholesterolemia and SCD, especially linked to Neimann–Pick C1. In any case, the rarity of the pathologies makes the management tailored on a case-by-case basis [62,63].

**Fatty Acid Metabolism Defects Associated with Hypertrophic Cardiomyopathy**

**Fatty acid metabolism defects**, particularly disorders affecting mitochondrial beta-oxidation, are a group of inherited metabolic diseases that can phenotypically mimic HCM, especially in pediatric populations. These disorders impair the ability of cardiomyocytes to utilize long-chain fatty acids, which are a primary energy source for the heart. The resulting **energy deficiency**, along with accumulation of toxic intermediates and lipid droplets, can trigger **myocardial hypertrophy**, fibrosis, and arrhythmias. Among these, **very long-chain acyl-CoA dehydrogenase deficiency** (VLCADD, OMIM 201475), **carnitine palmitoyltransferase II deficiency** (CPTII, OMIM 255110), and **multiple acyl-CoA dehydrogenase deficiency** (MADD, OMIM 231680) are most frequently associated with an HCM phenotype.

The clinical presentation is heterogeneous and may include hypoglycaemia, hepatomegaly, hypotonia, and recurrent rhabdomyolysis in addition to cardiomyopathy. The cardiac involvement can be severe, with concentric or asymmetric LV hypertrophy, early-onset heart failure, and risk of SCD. Diagnostic suspicion is raised by biochemical findings such as hypoketotic hypoglycaemia, elevated acylcarnitines, and muscle enzyme abnormalities, and confirmed by genetic testing and enzymatic assays. Treatment strategies include dietary fat restriction, medium-chain triglyceride supplementation, and emergency protocols during catabolic stress. Early recognition of these phenocopies is essential, as timely metabolic management can prevent progression of cardiac damage and improve outcomes [64,65].

## 7. Mitochondrial HCM

Mitochondrial diseases are multisystemic hereditary diseases with an estimated prevalence of 1 in 5000 newborns. About 50% of them are characterized by HCM, consisting of a small but still important percentage of all HCM. Isolated cardiac involvement is rare, while the main mitochondrial syndromes presenting with HCM are the following:

- Mitochondrial Encephalopathy, lactic acidosis and stroke-like episode syndrome (MELAS, OMIM 540000)
- Myoclonic Epilepsy with Ragged Red Fibers syndrome (MERRF, OMIM 545000)
- Leber Hereditary Optic Neuropathy syndrome (LHON, OMIM 535000)
- Chronic Progressive External Ophthalmoplegia syndrome (CPEO, OMIM 157640)
- Sengers syndrome (OMIM 212350) characterized by congenital cataract, HCM, skeletal myopathy, and lactic acidosis [66]

Mitochondrial diseases can be caused by both mitochondrial and nuclear DNA mutations. In the first case, transmission is matrilinear. Normal and mutant mitochondria can coexist (heteroplasmy) and pathology emerges when mutant mitochondria exceed a certain threshold in specific organs. In case of nuclear DNA mutations, transmission is autosomal dominant, autosomal recessive or X-linked. The great part of mitochondrial diseases is caused by nuclear DNA mutations, where there are many genes involved in cellular respiratory chain [67].

In this context, HCM consists of concentric symmetric hypertrophy with rare LVOT obstruction and common evolution towards heart failure. Arrhythmias can occur and complete atrioventricular block or ventricular tachycardia are to be feared as possible cause of SCD. Symptoms can start at any time from infancy to adulthood. Disease severity ranges from asymptomatic to disabling conditions and left ventricle hypertrophy onset is inversely related to prognosis [68].

Diagnosis is usually made by exclusion of other HCM etiologies when systemic manifestations of a mitochondrial disease are observed. In this process CMR is crucial to exclude other HCM etiologies, even if there is not a typical CMR for mitochondrial cardiomyopathies. Definitive diagnosis according to Bernier’s criteria need genetic tests, histological or biochemical examinations on cardiac samples [69]. However, these three tests are not easily feasible. Firstly, many different genes have been described as responsible for mitochondrial diseases, thus whole exome nuclear and mitochondrial sequencing should be performed for an accurate diagnosis. Secondly, even if electron microscopy allows us to recognize some mitochondrial abnormalities, histologic diagnostic criteria are not yet univocal. Thirdly, the biochemical tests to be performed for diagnosis are very specific and not always available [67].

Early-onset encephalopathy in infants, along with cardiomyopathy, is linked to a poorer prognosis. Prognosis is usually bad and the mortality rate before 30 years is high. Treatment consists of standard heart failure therapy and dietary supplements aiding mitochondrial biogenesis and function, such as creatine, carnitine, and coenzyme Q10. Furthermore, mitochondrial replacement therapies and gene therapy approaches are under investigation [70].

## 8. RASopathies

RASopathies are a group of multisystemic disorders resulting from mutations in genes associated with the RAS/MAPK (mitogen-activated protein kinase) signaling pathway. These genetic alterations lead to the constant activation of RAS proteins, causing aberrant and excessive cellular signaling, even without external stimuli [71,72]. RASopathies encompass a range of conditions, including neurofibromatosis type 1 (OMIM 162200), Noonan syndrome (OMIM 163950), Costello syndrome (OMIM 218040), cardio-facio-cutaneous syndrome (OMIM 115150) and Legius syndrome (OMIM 611431). Affected individuals typically experience dysfunction across multiple organ systems, presenting with craniofacial abnormalities, heart defects, skin, musculoskeletal, and eye irregularities, neurocognitive challenges, muscle weakness, and an elevated risk of cancer.

Some RASopathies, especially Noonan syndrome and Neurofibromatosis type 1 (NF1), could be associated with congenital heart disease (CHD), HCM, and dilated cardiomyopathy (DCM). In fact, RASopathies are a frequent cause of HCM in infancy and childhood, with the incidence varying, reaching its maximum in Noonan syndrome with multiple freckles (NSML) with a prevalence of approximately 80% of patients. Compared with non-syndromic patients, HCM in RASopathies has a more severe left ventricular hypertrophy (frequently asymmetrical) and a higher prevalence of LVOT obstruction, mitral valve anomalies, and biventricular hypertrophy. Even the septum morphology may differ among RASopathies, with a more sigmoid shape in NSML and a more biconvex shape in Noonan syndrome [73].

In patients with RASopathies, the ECG may show extreme right axis deviation due to biventricular hypertrophy and pseudo-infarction Q waves and prolonged QTc interval, together with atrial arrhythmias. In patients with RASopathies, HCM is a major determinant of clinical prognosis due to the risk of heart failure and SCD, and early diagnosis is crucial for improving the overall outcome of individuals with RASopathies. To date, no specific management therapies are available [71,72,73]. With the growth in genomic data, more details of genetic mutations associated with cardiac abnormalities could be identified and help in better understanding their physiopathology and treatment.

**Noonan syndrome**

Noonan syndrome, an autosomal dominant genetic disease, is classically associated with common congenital heart disease, especially pulmonary valve stenosis, in 50–60% of patients, and atrial septal defect. In almost 20% of patients, HCM is present and mostly diagnosed during infancy. A patient’s likelihood of HCM varies according to the gene mutated: in particular, mutations in the *RAF1* and *PTPN11* genes have been associated with an increased risk of HCM. A particular form of Noonan syndrome is the one with multiple freckles (NSML), formerly known as LEOPARD syndrome (lentigines, electrocardiographic abnormalities, ocular hypertelorism, pulmonic stenosis, abnormal male genitalia, retardation of growth and deafness), which is caused in 90% of cases by *PTPN11* mutations. At a histological level, hypertrophy of myocardial fibers comes together with focal interstitial fibrosis and no myocardial disarray. Among patients with RAS-associated HCM, the risk of SCD is greater than patients with HCM alone [71,72,73].

**Neurofibromatosis type 1**

Neurofibromatosis type 1, also an autosomal dominant genetic disorder, is a multisystem disease impacting the growth and function of multiple cells and organs. Cerebrovascular disease, pheochromocytomas, and cardiovascular disease are causes of premature death. Among cardiovascular implications, the most frequent are vasculopathy (aortic hypoplasia and intimal proliferation), hypertension, and congenital heart defects, especially pulmonary valve stenosis [71,72,73].

## 9. Neuromuscolar Diseases

Neuromuscular disorders often come with heart-related issues, including structural abnormalities and problems with electrical conduction. Although HCM is an uncommon feature of neuromuscular conditions, about 60% of cases in young adults and adults with HCM are linked to autosomal dominant mutations in sarcomeric genes. Other genetic conditions, including neuromuscular diseases, account for roughly 5–10% of HCM cases in adults. About neuromuscular diseases associated with HCM, X-linked recessive muscular dystrophies, myotonic dystrophies, and myofibrillar myopathies have to be considered. Also, rarer diseases like Barth syndrome, respiratory chain-related disorders, tRNA and rRNA-related disorders, mitochondrial depletion DNA syndromes, CoQ10 biosynthesis deficiency, limb girdle muscular dystrophy, facioscapulohumeral muscular dystrophy, congenital myopathy, and primary carnitine deficiency have been described associated with HCM [74,75].

**Friedreich’s ataxia**

In patients with Friedreich’s ataxia (OMIM 229300), HCM is common and generally progresses to concentric biventricular hypertrophy, with heart failure being the leading cause of death. Histologically, it is characterized by left ventricular cell hypertrophy, widespread fibrosis, and areas of myocardial necrosis. Echocardiograms typically reveal concentric left ventricle hypertrophy without obstruction of the LVOT, although eccentric hypertrophy may also be present. Diastolic function is usually mildly impaired, while global longitudinal strain is usually impaired. Unlike other diseases that cause concentric hypertrophy with a granular, sparkling texture (such as amyloidosis), atrial enlargement and pericardial effusion are rare in Friedreich’s ataxia. Left ventricle fibrosis has been observed and is associated with progressive thinning and dilation. In the late stages of Friedreich’s ataxia, patients can develop hypokinesia, reduced ejection fraction, atrial fibrillation, and other arrythmias. The use of idebenone has been described in these patients, but strong data are missing. Cardiac transplantation is not commonly performed due to the general condition of the patients [74,75].

**X-linked recessive muscular dystrophies**

The most common X-linked muscular dystrophies are Duchenne muscular dystrophy (DMD, OMIM 310200) and Becker muscular dystrophy (BMD, OMIM 300376). In DMD, mutations in the DMD gene result in a lack of functional dystrophin, while BMD is characterized by either shortened or reduced amounts of dystrophin. In BMD, cardiac involvement can appear before skeletal muscle weakness, with dilated cardiomyopathy being the typical cardiac outcome. However, a hypertrophic heart phenotype has been observed in some female carriers of dystrophinopathy, and cases of diastolic dysfunction followed by eccentric hypertrophy are also reported. Hypertrophic cardiomyopathy is rarely seen in BMD patients, but a few cases have been documented. In DMD patients, abnormal circumferential strain may be present even when the ejection fraction is normal and before symptoms appear. Cardiovascular complications are a major cause of illness and death in DMD patients. Although there is no specific treatment, therapy with ACE inhibitors may be beneficial, together with glucocorticoid in DMD. Some mutations in the *FHL1* gene, described in some families with X-linked Emery–Dreifuss muscular dystrophy, could be associated with HCM [74,75].

**Myotonic dystrophy type 1**

Myotonic dystrophy type 1 is a genetic multisystem disorder with autosomal dominant inheritance and incomplete penetrance. The primary clinical symptoms include myotonia and muscle weakness, though both the cardiovascular and respiratory systems are affected. Arrhythmias are the second leading cause of death in patients with myotonic dystrophy type 1 (DM-1, OMIM 160900), with many experiencing SCD. Cardiac involvement often includes concentric HCM, particularly in adults. Other structural abnormalities such as dilated cardiomyopathy (DCM) and left ventricular non-compaction (LVNC) or cardiac fatty infiltration have also been reported in these patients [74,75].

**Desminopathies**

Desminopathies are inherited myofibrillar myopathies caused by pathogenic variants in the *DES* gene, encoding desmin, a key intermediate filament protein in skeletal and cardiac muscle. Mutations disrupt the cytoskeletal network and its connection with the contractile apparatus, leading to desmin-positive protein aggregates, myofibrillar disarray, and progressive myocyte damage. Inheritance is most often autosomal dominant, although recessive forms with earlier and more severe presentations have been described. Clinically, desminopathies combine skeletal muscle weakness with cardiac involvement. The latter includes conduction system disease (atrioventricular or bundle branch block), supraventricular and ventricular arrhythmias, and cardiomyopathy, which may be dilated, restrictive, or, less frequently, hypertrophic, thus acting as an HCM phenocopy. Age at onset is highly variable, ranging from adolescence to late adulthood, with most patients presenting in early to mid-adulthood. Echocardiography and CMR may show concentric or asymmetric hypertrophy and mid-wall or patchy LGE, reflecting myocardial fibrosis. Muscle biopsy typically shows characteristic desmin-positive cytoplasmic aggregates, Z-line streaming, and myofibrillar disruption, but in current practice diagnosis is increasingly based on genetic testing. The management is mainly supportive, focusing on heart failure therapy, rhythm control, and timely pacemaker or ICD implantation, together with family screening [76,77,78].

## 10. HCM Phenocopies Differential Diagnosis: A Practical Approach

Considering that non-sarcomeric HCM phenocopies are relatively common overall, it is crucial to diagnose these conditions at an early stage, as their natural history, management, and prognosis differ significantly from that of HCM with sarcomeric mutations.

Diagnosis suspicion should be guided by familial history and clinical red flags, which are different for each hypertrophic phenocopy. However, absence of signs and symptoms does not preclude diagnosis, and unfortunately SCD can be the first HCM manifestation.

ECG can support HCM suspicion and rhythm alterations are frequent, especially atrial fibrillation.
Echocardiography is the first-level imaging exam for HCM diagnosis, showing left ventricle hypertrophy and diastolic dysfunction as main features. Other typical findings are left atrium enlargement, systolic disfunction evaluated by global longitudinal strain (GLS), and LVOT obstruction due to systolic anterior motion (SAM) of the mitral valve.
Once a hypertrophic cardiomyopathy phenotype has been recognized, CMR can confirm morphological characteristics with higher accuracy and reproducibility than echocardiography and it is an ideal tool for differential diagnosis of HCM phenocopies thanks to a multiparametric tissue characterization.
Genetic tests are crucial for many HCM phenocopies diagnosis and a genetic panel should be considered, especially in severe diseases with early onset.
Furthermore, specific lab or imaging studies may be needed for specific HCM phenocopies: for example, for amyloidosis subtype diagnosis, serum and urine protein electrophoresis with immunofixation and free light chain dosage and bone scintigraphy with 99mTc-DPD/PYP/HMDP are mandatory (Figure 3).

**Personal history**
Age of onset

In the diagnostic pathway of HCM phenocopies, it is crucial to focus on the main differences. The age of first presentation is a good starting point because, for example, inborn errors of metabolism or congenital syndromes are more common at a young age, while wild-type ATTR is typical of elderly people (Table 1).

Symptoms and physical examination

Systemic symptoms other than those due to cardiac involvement can be the first element which the patient presents for medical attention. Indeed, hypertrophic cardiomyopathies are a common element of multisystemic diseases. Some examples of signs and symptoms associated with a specific HCM phenocopy are presented in Table 2.

**Family history/Inheritance**

Many HCM phenocopies are associated with hereditary disorders. Constructing a family pedigree is therefore crucial to identify potential patterns of inheritance, while also considering the possibility of de novo mutations, which can further guide the diagnostic process. Table 3 summarizes the pattern of inheritance of the most common HCM phenocopies.

**Electrocardiogram**

An ECG is often the first abnormal finding suggestive of HCM. It is important to interpret the ECG in the context of the clinical presentation and to compare it with imaging findings, such as echocardiography and CMR. Some ECG features that may help guide the diagnosis of HCM phenocopies are presented in Table 4.

**Table 3:** Patterns of inheritance of the main HCM phenocopies. HCM: hypertrophic cardiomyopathy.

	Autosomal Dominant	Autosomal Recessive	X-Linked	Matrilinear
**Sarcomeric HCM**	X			
**Familial transthyretin-related amyloidosis**	X			
**Fabry Disease**			X	
**Pompe disease**		X		
**Danon disease**			X	
**Forbes disease**		X		
***PRKAG2* cardiomyopathy**	X			
**Hurler syndrome**		X		
**Gaucher disease**		X		
**Niemann–Pick disease**		X		
**Fatty acid metabolism defects**		X		
**Mitochondrial cardiomyopathies**	X	X	X	X
**Noonan syndrome**	X			
**Neurofibromatosis type 1**	X			
**Friedreich’s ataxia**		X		
**Desminopathies**	X	X		
**Mitochondrial cardiomyopathies**	X	X	X	X
**Duchenne dystrophy**			X	
**Becker dystrophy**			X	
**Myotonic dystrophy type 1**	X			

**Table 4:** Electrocardiographic abnormalities suggest specific HCM phenocopy diagnosis. HCM: hypertrophic cardiomyopathy.

ECG Abnormality	Diagnoses Suspected
**Short PR interval**	Fabry disease, mitochondrial cardiomyopathies, Pompe disease, Hurler syndrome
**Long PR interval**	Sarcomeric HCM, Friedreich’s ataxia, neurofibromatosis type 1, myotonic dystrophy type 1
**Advanced AV blocks**	Amyloidosis, Fabry disease, *PRKAG2*, mitochondrial cytopathies, Danon disease
**High QRS voltages**	Sarcomeric HCM, *PRKAG2*, Forbes disease, Pompe disease, Danon disease, Hurler syndrome
**Low QRS voltage (or ECG voltage to hypertrophy mismatch)**	Amyloidosis, Friedreich’s ataxia
**Ventricular pre-excitation (pattern WPW-like)**	Danon disease, Pompe disease, *PRKAG2*
**Pseudonecrosis pattern (pathological Q waves)**	Sarcomeric HCM, Amyloidosis, Duchenne/Becker dystrophy
**Prolonged QT**	Mitochondrial cardiomyopathies, Fatty acid metabolism defects, Myotonic dystrophy type 1
**Inverted T waves**	Sarcomeric HCM, Fabry disease, mitochondrial cardiomyopathies, Noonan syndrome, Friedreich’s ataxia
**Ventricular arrhythmias**	Sarcomeric HCM, Danon disease, *PRKAG2*, Fatty acid metabolism defects, mitochondrial cardiomyopathies

**Echocardiography**

Transthoracic echocardiography remains the first-line imaging exam for the identification and assessment of HCM phenocopies. Several red flags should be recognized to avoid missing or misdiagnosing HCM phenocopies, as summarized in Table 5.

**Cardiac magnetic resonance**

Among imaging techniques, CMR is the most important for the differential diagnosis of HCM-related cardiomyopathies, particularly due to its ability to provide non-invasive tissue characterization of the myocardium [11]. Native T1 is sensitive to cellular damage, thus it is abnormal especially in storage diseases. Gadolinium is a contrast agent with extracellular distribution; thus, LGE can explore extracellular space, differentiating between HCM due to storage (as Fabry disease) or extracellular infiltrates (as amyloidosis). Furthermore, extracellular space itself can be derived as a percentage through native T1 and post-contrast T1 comparison. The latter is higher than normal in infiltrative diseases and lower in storage diseases. Finally, T2 mapping is sensitive to water and thus to tissue edema as an expression of segmental or diffuse myocardial damage. Some typical CMR findings could be related to a specific HCM phenocopy, as presented in Table 6.

**Genetic testing**

Many HCM phenocopies are caused by inherited or acquired genetic mutations. The identification of such mutations is an integral part of the diagnostic process and is often required for a definitive diagnosis and/or for initiating specific therapy. Although general gene panels are available, especially for rarer forms, the choice of which mutations to investigate should be guided by clinical suspicion and by the ECG, echocardiographic, and CMR features of the case. Table 7 summarizes the main genetic mutations associated with HCM phenocopies. Many mutations in these genes show incomplete penetrance, and complex phenotypes are often the result of more than one mutation coexisting in the same individual. An especially relevant topic is the study of genotype–phenotype correlations, particularly in sarcomeric HCM, although robust data in this field are still lacking. Moreover, the mechanisms by which many of these variants lead to sarcomeric dysfunction are only partially understood [79].

**Phenocopy-specific diagnostic tests**

The most recent ESC guidelines on cardiomyopathies advise performing routine laboratory tests in all patients with suspected or confirmed cardiomyopathy in order to investigate the underlying etiology, evaluate disease severity, and aid in the identification of extracardiac involvement and secondary organ dysfunction (class I, level C). Among first-line lab tests, NT-proBNP and troponin are especially useful. Furthermore, second-level laboratory assessments or specific imaging exams are recommended in patients with cardiomyopathy and associated extracardiac signs to facilitate the recognition of potential metabolic or syndromic conditions, following specialist consultation (class IIa, level C) [3]. Useful specific first- and second-level tests for the main HCM phenocopies are presented in Table 8.

## 11. Conclusions

In conclusion, the classification and differential diagnosis of HCM phenocopies require a multidisciplinary approach, combining clinical evaluation, imaging, and genetic testing. HCM and cardiac amyloidosis account for nearly 80% of all HCM phenocopies; however, many other diseases can cause cardiac hypertrophy. Therefore, awareness and recognition of HCM phenocopies in clinical practice is crucial to enhance patient care and to optimize therapeutic strategies with a major impact on patients’ quality of life and prognosis.

## Figures and Tables

**Table 1:** Typical age of onset of main HCM phenocopies symptoms. HCM: hypertrophic cardiomyopathy.

	Neonate/Childhood	Adolescence/ Young Adult	Middle Age	Elderly
**Sarcomeric HCM**		X	X	
**Cardiac amyloidosis**			X	X
**Fabry Disease**	X	X	X	
**Pompe disease**	X	X		
**Danon disease**		X		
**Forbes disease**	X			
***PRKAG2* cardiomyopathy**		X		
**Hurler syndrome**	X			
**Gaucher disease**	X	X		
**Niemann–Pick disease**	X	X	X	X
**Fatty acid metabolism defects**	X			
**Mitochondrial cardiomyopathies**	X			
**Noonan syndrome**	X			
**Neurofibromatosis type 1**	X			
**Friedreich’s ataxia**	X			
**Desminopathies**		X	X	
**Duchenne dystrophy**	X			
**Becker dystrophy**	X	X		
**Myotonic dystrophy type 1**		X	X	

**Table 2:** Signs and symptoms suggesting specific HCM phenocopies. ATTR: transthyretin amyloidosis; HCM: hypertrophic cardiomyopathy.

Signs and Symptoms	Diagnoses Suspected
**Sensorineural deafness**	Fabry disease, mitochondrial diseases, Noonan syndrome
**Visual disturbances**	ATTR, Fabry disease, Danon disease, mitochondrial diseases, Niemann–Pick disease
**Paresthesia/sensory abnormalities/neuropathic pain**	Amyloidosis, Fabry disease
**Neuromotor disorders (ataxia, dysphagia, dysarthria)**	Friedreich’s ataxia, Niemann–Pick disease
**Learning disabilities, intellectual disability**	Mitochondrial diseases, Noonan syndrome, Danon disease,
**Gait impairment**	Friedreich’s ataxia, Niemann–Pick disease
**Carpal tunnel syndrome (especially if bilateral)**	ATTR
**Muscle weakness**	Glycogen storage diseases, mitochondrial diseases, neuromuscular disorders
**Hepatosplenomegaly**	Niemann–Pick disease
**Palpebral ptosis**	Mitochondrial diseases, myotonic dystrophy
**Lentigines or café au lait spots**	Fabry disease, Noonan syndrome
**Angiokeratoma**	Fabry disease

**Table 5:** Echocardiographic red flags related to HCM phenocopies. HCM: hypertrophic cardiomyopathy.

Echo Finding	Diagnoses Suspected
**Increased inter-atrial septum thickness**	Sarcomeric HCM, amyloidosis
**Severe left atrial enlargement**	Sarcomeric HCM, amyloidosis, mitochondrial cardiomyopathies
**Concentric left ventricular hypertrophy**	Amyloidosis, Fabry disease, *PRKAG2* cardiomyopathy, mitochondrial cardiomyopathies
**Asymmetric septal hypertrophy**	Sarcomeric HCM, Noonan syndrome, *PRKAG2* cardiomyopathy
**Apical aneurysms**	Sarcomeric HCM (rare), Friedreich’s ataxia
**Ground-glass aspect of ventricular myocardium**	Amyloidosis
**Early alterations of global longitudinal strain**	Sarcomeric HCM, amyloidosis, Fabry disease, Danon disease, *PRKAG2* cardiomyopathy, mitochondrial diseases
**Reduced longitudinal strain with apical sparing**	Amyloidosis
**Restrictive diastolic filling pattern**	Amyloidosis, Danon disease, Friedreich’s ataxia, mitochondrial cardiomyopathies
**Progressive left ventricular dilatation**	Duchenne dystrophy, Becker dystrophy, myotonic dystrophy type 1, mitochondrial cardiomyopathies, fatty acid metabolism defects
**Increased valvular thickening**	Amyloidosis, Fabry disease, Hurler syndrome
**Increased right ventricle free wall thickness**	Amyloidosis, Fabry disease
**Pericardial effusion**	Amyloidosis

**Table 6:** Tissue characterization by CMR findings related to HCM phenocopies. HCM: hypertrophic cardiomyopathy.

CMR Finding	Diagnoses Suspected
**Diffuse T1 shortening**	Fabry disease
**Diffuse T1 elevation**	Amyloidosis
**Diffuse subendocardial LGE**	Amyloidosis
**Mid-wall/patchy LGE**	Sarcomeric HCM, Danon disease, *PRKAG2* cardiomyopathy, Friedreich’s ataxia
**Posterolateral LGE**	Fabry disease
**No LGE or minimal LGE**	Fabry disease (early), *PRKAG2* cardiomyopathy (early)
**Elevated ECV**	Amyloidosis, Fabry disease (advanced)
**Abnormal T2 signal (edema/inflammation)**	Pompe disease, Danon disease, Duchenne/Becker dystrophy
**Fatty infiltration**	Danon disease, Pompe disease, *PRKAG2* cardiomyopathy
**Myocardial fibrosis**	Sarcomeric HCM, Danon disease, Friedreich’s ataxia, Duchenne/Becker dystrophy

**Table 7:** Key causative genes in the main HCM phenocopies. HCM: hypertrophic cardiomyopathy; mtDNA: mitochondrial DNA.

	Main Gene(s) Involved	Notes
**Sarcomeric HCM**	*MYH7*, *MYBPC3*, *TNNT2*, *TNNI3*, *TPM1*	Sarcomeric protein mutations
**ATTRm**	*TTR*	Transthyretin gene
**AL**	*IGH* locus, *MYD 88*, *TP53*	Acquired plasma cell disorder
**Fabry Disease**	*GLA*	Alpha-galactosidase A deficiency
**Pompe disease**	*GAA*	Acid alpha-glucosidase deficiency
**Danon disease**	*LAMP2*	Lysosomal-associated membrane protein 2
**Forbes disease (GSD III)**	*AGL*	Glycogen debranching enzyme deficiency
***PRKAG2* cardiomyopathy**	*PRKAG2*	AMPK regulatory subunit
**Hurler syndrome**	*IDUA*	Alpha-L-iduronidase deficiency
**Gaucher disease**	*GBA*	Beta-glucocerebrosidase deficiency
**Niemann–Pick disease**	*SMPD1*, *NPC1*, *NPC2*	Sphingomyelinase/lysosomal transport defects
**Fatty acid metabolism defects**	*ACADVL*, *CPT2*, *HADHA*, *HADHB*	Fatty acid oxidation defects
**Mitochondrial** **cardiomyopathies**	*MT-TL1*, *MT-ND1*	mtDNA or nuclear genes
**Noonan syndrome**	*PTPN11*, *SOS1*, *RAF1*, *KRAS*	RAS/MAPK pathway genes
**Neurofibromatosis type 1**	*NF1*	Neurofibromin
**Friedreich’s ataxia**	*FXN*	Frataxin gene
**Duchenne dystrophy**	*DMD*	Dystrophin gene
**Becker dystrophy**	*DMD*	Dystrophin gene
**Myotonic dystrophy type 1**	*DMPK*	Dystrophia myotonica protein kinase gene

**Table 8:** Specific first- and second-level laboratory and imaging tests for the main HCM phenocopy diagnoses. AL: light chain amyloidosis; ATTR: transthyretin amyloidosis; CK: creatine kinase; CPK: creatine phosphokinase; CSF: cerebro-spinal fluid; HCMs: hypertrophic cardiomyopathies; PET: positron emission tomography.

	Specific First-Level Lab Tests	Second-Level Lab and Imaging Tests
**Sarcomeric HCM**		CPK if myopathic suspicion
**Cardiac amyloidosis**	serum and urine protein electrophoresis with immunofixation, free light chains	Bone scintigraphy, cardiac/salivary glands/fat biopsy with Congo red, PET (investigated for AL vs. ATTR diagnosis)
**Fabry Disease**	-	α-galactosidase A activity in men, plasma Lyso-Gb-3 in women
**Pompe disease**	CK	α-glucosidase A activity
**Danon disease**	CK, liver enzymes	α-glucosidase A activity, skeletal muscle biopsy
**Forbes disease**	CK, liver enzymes, glucose, lactate	Debranching enzyme activity
***PRKAG2* cardiomyopathy**	CK	-
**Hurler syndrome**	CK, liver enzymes	Urinary GAGs, α-L-iduronidase activity,
**Gaucher disease**	ferritin, liver enzymes	β-glucocerebrosidase activity, chitotriosidase,
**Niemann–Pick disease**	liver enzymes	Sphingomyelinase activity (types A/B), oxysterols (type C),
**Fatty acid metabolism defects**	CK, glucose, lactate	Acylcarnitine profile, enzyme assays
**Mitochondrial cardiomyopathies**	CK, lactate, urine myoglobin	Plasma/CSF lactate and pyruvate
**Noonan syndrome**	-	-
**Neurofibromatosis type 1**	-	-
**Friedreich’s ataxia**	glucose	glucose tolerance test
**Duchenne dystrophy**	CK	dystrophin analysis
**Becker dystrophy**	CK	dystrophin analysis
**Myotonic dystrophy type 1**	CK, glucose, liver enzymes	

## Data Availability

No new data were created or analyzed in this study.

## Abbreviations

The following abbreviations are used in this manuscript:

AGAL-A	α-galactosidase A
AL	Light-chain amyloidosis
ATTR	Transthyretin amyloidosis
ATTRv	Variant transthyretin amyloidosis
ATTRwt	Wild-type transthyretin amyloidosis
BMD	Becker muscular dystrophy
CMR	Cardiac magnetic resonance
DMD	Duchenne muscular dystrophy
ECG	Electrocardiogram
ERT	Enzyme replacement therapy
*FHL-1*	Four and Half LIM domain-1
GSD	Glycogen storage disorder
HCM	Hypertrophic cardiomyopathy/cardiomyopathies
LGE	Late gadolinium enhancement
LVOT	Left ventricle outflow tract
MPS	Mucopolysaccharidoses
SCD	Sudden cardiac death

## References

[1] Vio R., Angelini A., Basso C., Cipriani A., Zorzi A., Melacini P., Thiene G., Rampazzo A., Corrado D., Calore C. (2021). Hypertrophic cardiomyopathy and primary restrictive cardiomyopathy: Similarities, differences and phenocopies. J. Clin. Med..

[2] Arbustini E., Narula N., Tavazzi L., Serio A., Grasso M., Favalli V., Bellazzi R., Tajik J.A., Bonow R.O., Fuster V. (2014). The MOGE(S) classification of cardiomyopathy for clinicians. J. Am. Coll. Cardiol..

[3] Arbelo E., Protonotarios A., Gimeno J.R., Arbustini E., Barriales-Villa R., Basso C., Bezzina C.R., Biagini E., Blom N.A., de Boer R.A. (2023). 2023 ESC Guidelines for the management of cardiomyopathies: Developed by the task force on the management of cardiomyopathies of the European Society of Cardiology (ESC). Eur. Heart J..

[4] Ciarambino T., Menna G., Sansone G., Giordano M. (2021). Cardiomyopathies: An Overview. Int. J. Mol. Sci..

[5] McKenna W.J., Judge D.P. (2021). Epidemiology of the inherited cardiomyopathies. Nat. Rev. Cardiol..

[6] Limongelli G., Masarone D., Verrengia M., Gravino R., Salerno G., Castelletti S., Rubino M., Marrazzo T., Pisani A., Cecchi F. (2018). Diagnostic Clues for the Diagnosis of Nonsarcomeric Hypertrophic Cardiomyopathy (Phenocopies): Amyloidosis, Fabry Disease, and Mitochondrial Disease. J. Cardiovasc. Echogr..

[7] Rapezzi C., Arbustini E., Caforio A.L.P., Charron P., Gimeno-Blanes J., Heliö T., Linhart A., Mogensen J., Pinto Y., Ristic A. (2013). Diagnostic work-up in cardiomyopathies: Bridging the gap between clinical phenotypes and final diagnosis. A position statement from the ESC Working Group on Myocardial and Pericardial Diseases. Eur. Heart J..

[8] Baggiano A., Del Torto A., Guglielmo M., Muscogiuri G., Fusini L., Babbaro M., Collevecchio A., Mollace R., Scafuri S., Mushtaq S. (2020). Role of CMR mapping techniques in cardiac hypertrophic phenotype. Diagnostics.

[9] Sankaranarayanan R., Fleming E.J., Garratt C.J. (2013). Mimics of Hypertrophic Cardiomyopathy—Diagnostic Clues to Aid Early Identification of Phenocopies. Arrhythmia Electrophysiol. Rev..

[10] Ommen S.R., Mital S., Burke M.A., Day S.M., Deswal A., Elliott P., Evanovich L.L., Hung J., Joglar J.A., Kantor P. (2020). 2020 AHA/ACC Guideline for the Diagnosis and Treatment of Patients With Hypertrophic Cardiomyopathy: A Report of the American College of Cardiology/American Heart Association Joint Committee on Clinical Practice Guidelines. J. Am. Coll. Cardiol..

[11] Licordari R., Trimarchi G., Teresi L., Restelli D., Lofrumento F., Perna A., Campisi M., de Gregorio C., Grimaldi P., Calabrò D. (2023). Cardiac Magnetic Resonance in HCM Phenocopies: From Diagnosis to Risk Stratification and Therapeutic Management. J. Clin. Med..

[12] Brito D., Miltenberger-Miltenyi G., Pereira S.V., Silva D., Diogo A.N., Madeira H. (2012). Sarcomeric hypertrophic cardiomyopathy: Genetic profile in a Portuguese population. Rev. Port. Cardiol..

[13] Marian A.J., Braunwald E. (2017). Hypertrophic Cardiomyopathy: Genetics, Pathogenesis, Clinical Manifestations, Diagnosis, and Therapy. Circ. Res..

[14] Girolami F., Ho C.Y., Semsarian C., Baldi M., Will M.L., Baldini K., Torricelli F., Yeates L., Cecchi F., Ackerman M.J. (2010). Clinical features and outcome of hypertrophic cardiomyopathy associated with triple sarcomere protein gene mutations. J. Am. Coll. Cardiol..

[15] Ingles J., Doolan A., Chiu C., Seidman J., Seidman C., Semsarian C. (2005). Compound and double mutations in patients with hypertrophic cardiomyopathy: Implications for genetic testing and counselling. J. Med. Genet..

[16] Zito C., Longobardo L., Citro R., Galderisi M., Oreto L., Carerj M.L., Manganaro R., Cusmà-Piccione M., Todaro M.C., Di Bella G. (2018). Ten Years of 2D Longitudinal Strain for Early Myocardial Dysfunction Detection: A Clinical Overview. BioMed. Res. Int..

[17] Maron B.J., Desai M.Y., Nishimura R.A., Spirito P., Rakowski H., Towbin J.A., Rowin E.J., Maron M.S., Sherrid M.V. (2022). Diagnosis and Evaluation of Hypertrophic Cardiomyopathy: JACC State-of-the-Art Review. J. Am. Coll. Cardiol..

[18] Tucholski T., Cai W., Gregorich Z.R., Bayne E.F., Mitchell S.D., McIlwain S.J., de Lange W.J., Wrobbel M., Karp H., Hite Z. (2020). Distinct hypertrophic cardiomyopathy genotypes result in convergent sarcomeric proteoform profiles revealed by top-down proteomics. Proc. Natl. Acad. Sci. USA.

[19] Maron B.J., Desai M.Y., Nishimura R.A., Spirito P., Rakowski H., Towbin J.A., Dearani J.A., Rowin E.J., Maron M.S., Sherrid M.V. (2022). Management of Hypertrophic Cardiomyopathy: JACC State-of-the-Art Review. J. Am. Coll. Cardiol..

[20] Rubin J., Maurer M.S. (2020). Cardiac Amyloidosis: Overlooked, Underappreciated, and Treatable. Annu. Rev. Med..

[21] Aimo A., Merlo M., Porcari A., Georgiopoulos G., Pagura L., Vergaro G., Sinagra G., Emdin M., Rapezzi C. (2022). Redefining the epidemiology of cardiac amyloidosis. A systematic review and meta-analysis of screening studies. Eur. J. Heart Fail..

[22] Martinez-Naharro A., Hawkins P.N., Fontana M. (2018). Cardiac amyloidosis. Clin. Med..

[23] Maurer M.S., Bokhari S., Damy T., Dorbala S., Drachman B.M., Fontana M., Grogan M., Kristen A.V., Lousada I., Nativi-Nicolau J. (2019). Expert Consensus Recommendations for the Suspicion and Diagnosis of Transthyretin Cardiac Amyloidosis. Circ. Heart Fail..

[24] Siddiqi O.K., Ruberg F.L. (2018). Cardiac amyloidosis: An update on pathophysiology, diagnosis, and treatment. Trends Cardiovasc. Med..

[25] de Gregorio C., Trimarchi G., Faro D.C., Poleggi C., Teresi L., De Gaetano F., Zito C., Lofrumento F., Koniari I., Licordari R. (2024). Systemic Vascular Resistance and Myocardial Work Analysis in Hypertrophic Cardiomyopathy and Transthyretin Cardiac Amyloidosis with Preserved Left Ventricular Ejection Fraction. J. Clin. Med..

[26] Teresi L., Trimarchi G., Liotta P., Restelli D., Licordari R., Carciotto G., Francesco C., Crea P., Dattilo G., Micari A. (2024). Electrocardiographic Patterns and Arrhythmias in Cardiac Amyloidosis: From Diagnosis to Therapeutic Management—A Narrative Review. J. Clin. Med..

[27] Cersosimo A., Bonelli A., Lombardi C.M., Moreo A., Pagnesi M., Tomasoni D., Arabia G., Vizzardi E., Adamo M., Farina D. (2023). Multimodality imaging in the diagnostic management of concomitant aortic stenosis and transthyretin-related wild-type cardiac amyloidosis. Front. Cardiovasc. Med..

[28] Monte I.P., Faro D.C., Trimarchi G., de Gaetano F., Campisi M., Losi V., Teresi L., Di Bella G., Tamburino C., de Gregorio C. (2023). Left Atrial Strain Imaging by Speckle Tracking Echocardiography: The Supportive Diagnostic Value in Cardiac Amyloidosis and Hypertrophic Cardiomyopathy. J. Cardiovasc. Dev. Dis..

[29] Gillmore J.D., Maurer M.S., Falk R.H., Merlini G., Damy T., Dispenzieri A., Wechalekar A.D., Berk J.L., Quarta C.C., Grogan M. (2016). Nonbiopsy diagnosis of cardiac transthyretin amyloidosis. Circulation.

[30] Genovesi D., Vergaro G., Giorgetti A., Marzullo P., Scipioni M., Santarelli M.F., Pucci A., Buda G., Volpi E., Emdin M. (2021). [18F]-Florbetaben PET/CT for Differential Diagnosis Among Cardiac Immunoglobulin Light Chain, Transthyretin Amyloidosis, and Mimicking Conditions. JACC Cardiovasc. Imaging.

[31] Aimo A., Rapezzi C., Perfetto F., Cappelli F., Palladini G., Obici L., Merlini G., Di Bella G., Serenelli M., Zampieri M. (2021). Quality of life assessment in amyloid transthyretin (ATTR) amyloidosis. Eur. J. Clin. Investig..

[32] Alberto A., Lucio T., Vincenzo C., Lisa P.A., Martina N., Silvia S., Baraglia A.C., Obici L., Palladini G., Ponti L. (2024). Patient-reported outcome measures for transthyretin cardiac amyloidosis: The ITALY study. Amyloid.

[33] Garcia-Pavia P., Rapezzi C., Adler Y., Arad M., Basso C., Brucato A., Burazor I., Caforio A.L.P., Damy T., Eriksson U. (2021). Diagnosis and treatment of cardiac amyloidosis: A position statement of the ESC Working Group on Myocardial and Pericardial Diseases. Eur. Heart J..

[34] Maurer M.S., Schwartz J.H., Gundapaneni B., Elliott P.M., Merlini G., Waddington-Cruz M., Kristen A.V., Grogan M., Witteles R., Damy T. (2018). Tafamidis Treatment for Patients with Transthyretin Amyloid Cardiomyopathy. N. Engl. J. Med..

[35] De Francesco P.N., Mucci J.M., Ceci R., Fossati C.A., Rozenfeld P.A. (2013). Fabry disease peripheral blood immune cells release inflammatory cytokines: Role of globotriaosylceramide. Mol. Genet. Metab..

[36] Militaru S., Jurcuț R., Adam R., Roşca M., Ginghina C., Popescu B.A. (2019). Echocardiographic features of Fabry cardiomyopathy-Comparison with hypertrophy-matched sarcomeric hypertrophic cardiomyopathy. Echocardiography.

[37] Pieroni M., Moon J.C., Arbustini E., Barriales-Villa R., Camporeale A., Vujkovac A.C., Elliott P.M., Hagege A., Kuusisto J., Linhart A. (2021). Cardiac Involvement in Fabry Disease: JACC Review Topic of the Week. J. Am. Coll. Cardiol..

[38] Gümüş E., Özen H. (2023). Glycogen storage diseases: An update. World, J. Gastroenterol..

[39] Ellingwood S.S., Cheng A. (2018). Biochemical and clinical aspects of glycogen storage diseases. J. Endocrinol..

[40] Martínez M., Romero M., Guereta L., Cabrera M., Regojo R., Albajara L., Couce M.L., Pipaon M.S. (2017). Infantile-onset Pompe disease with neonatal debut: A case report and literature review. Medicine.

[41] Arad M., Maron B.J., Gorham J.M., Johnson W.H.J., Saul J.P., Perez-Atayde A.R., Spirito P., Wright G.B., Kanter R.J., Seidman C.E. (2005). Glycogen storage diseases presenting as hypertrophic cardiomyopathy. N. Engl. J. Med..

[42] Stevens D., Milani-Nejad S., Mozaffar T. (2022). Pompe Disease: A Clinical, Diagnostic, and Therapeutic Overview. Curr. Treat. Options Neurol..

[43] Labella B., Piccinelli S.C., Risi B., Caria F., Damioli S., Bertella E., Poli L., Padovani A., Filosto M. (2023). A Comprehensive Update on Late-Onset Pompe Disease. Biomolecules.

[44] Scheffers L., Kok R., Berg L.v.D., Hout J.v.D., Boersma E., van Capelle C., Helbing W., van der Ploeg A., Koopman L. (2023). Effects of enzyme replacement therapy on cardiac function in classic infantile Pompe disease. Int. J. Cardiol..

[45] Endo Y., Furuta A., Nishino I. (2015). Danon disease: A phenotypic expression of LAMP-2 deficiency. Acta Neuropathol..

[46] Cheng Z., Fang Q. (2012). Danon disease: Focusing on heart. J. Hum. Genet..

[47] Hong K.N., Eshraghian E.A., Arad M., Argirò A., Brambatti M., Bui Q., Caspi O., de Frutos F., Greenberg B., Ho C.Y. (2023). International Consensus on Differential Diagnosis and Management of Patients With Danon Disease: JACC State-of-the-Art Review. J. Am. Coll. Cardiol..

[48] https://www.orpha.net/en/disease/detail/366.

[49] Kishnani P.S., Austin S.L., Arn P., Bali D.S., Boney A., Case L.E., Chung W.K., Desai D.M., El-Gharbawy A., Haller R. (2010). Glycogen Storage Disease Type III diagnosis and management guidelines. Genet. Med..

[50] Lee P.J., Deanfield J.E., Burch M., Baig K., McKenna W.J., Leonard J.V. (1997). Comparison of the functional significance of left ventricular hypertrophy in hypertrophic cardiomyopathy and glycogenosis type III. Am. J. Cardiol..

[51] Schreuder A., Rossi A., Grunert T. (2022). Glycogen storage disease type III. GeneReviews.

[52] Gollob M.H., Green M.S., Tang A.S.-L., Gollob T., Karibe A., Hassan A.-S., Ahmad F., Lozado R., Shah G., Fananapazir L. (2001). Identification of a gene responsible for familial Wolff-Parkinson-White syndrome. N. Engl. J. Med..

[53] Maron B.J., Maron M.S. (2020). PRKAG2 Glycogen Storage Disease Cardiomyopathy: Out of the Darkness and Into the Light. J. Am. Coll. Cardiol..

[54] Dos Santos Neto D.A., Souza Neto I.D., Barbosa A.P., Sternick E.B., Pena J.L.B. (2024). Echocardiographic Findings in Children of Patients Diagnosed with PRKAG2 Syndrome. Arq. Bras. Cardiol..

[55] Lopez-Sainz A., Dominguez F., Lopes L.R., Ochoa J.P., Barriales-Villa R., Climent V., Linschoten M., Tiron C., Chiriatti C., Marques N. (2020). Clinical Features and Natural History of PRKAG2 Variant Cardiac Glycogenosis. J. Am. Coll. Cardiol..

[56] Banankhah P., Fishbein G.A., Dota A., Ardehali R. (2018). Cardiac manifestations of PRKAG2 mutation. BMC Med. Genet..

[57] Sestito S., Rinninella G., Rampazzo A., D’avanzo F., Zampini L., Santoro L., Gabrielli O., Fiumara A., Barone R., Volpi N. (2022). Cardiac involvement in MPS patients: Incidence and response to therapy in an Italian multicentre study. Orphanet J. Rare Dis..

[58] Pillai N.R., Elsbecker S.A., Gupta A.O., Lund T.C., Orchard P.J., Braunlin E. (2023). Hematopoietic cell transplantation for Mucopolysaccharidosis I in the presence of decreased cardiac function. Mol. Genet. Metab..

[59] Braunlin E.A., Harmatz P.R., Scarpa M., Furlanetto B., Kampmann C., Loehr J.P., Ponder K.P., Roberts W.C., Rosenfeld H.M. (2011). Cardiac disease in patients with mucopolysaccharidosis: Presentation, diagnosis and management. J. Inherit. Metab. Dis..

[60] Lazea C., Bucerzan S., Al-Khzouz C., Zimmermann A., Vesa Ș.C., Nașcu I., Creț V., Crișan M., Asăvoaie C., Miclea D. (2021). Cardiac Manifestations in a Group of Romanian Patients with Gaucher Disease Type 1 (a Monocentric Study. Diagnostics.

[61] Lee E., Katz R., Choi A. (2021). A Rare Case of Cardiac Involvement in Gaucher’s Disease. J. Am. Coll. Cardiol..

[62] Zhao W., Zhang Q., Wang J., Yu H., Zhen X., Li L., Qu Y., He Y., Zhang J., Li C. (2022). Novel Indel Variation of NPC1 Gene Associates With Risk of Sudden Cardiac Death. Front. Genet..

[63] Geberhiwot T., Wasserstein M., Wanninayake S., Bolton S.C., Dardis A., Lehman A., Lidove O., Dawson C., Giugliani R., Imrie J. (2023). Consensus clinical management guidelines for acid sphingomyelinase deficiency (Niemann–Pick disease types A., B and A/B). Orphanet J. Rare Dis..

[64] Merritt J.L., Norris M., Kanungo S. (2018). Fatty Acid Oxidation disorders. Ann. Transl. Med..

[65] Elizondo G., Saini A., de Alba C.G., Gregor A., Harding C.O., Gillingham M.B., Vinocur J.M. (2024). Cardiac phenotype in adolescents and young adults with long-chain 3-hydroxyacyl CoA dehydrogenase (LCHAD) deficiency. Genet. Med..

[66] Mazzaccara C., Mirra B., Barretta F., Caiazza M., Lombardo B., Scudiero O., Tinto N., Limongelli G., Frisso G. (2021). Molecular epidemiology of mitochondrial cardiomyopathy: A search among mitochondrial and nuclear genes. Int. J. Mol. Sci..

[67] Takeda A. (2020). Mitochondrial cardiomyopathy. J. Pediatr. Cardiol. Card. Surg..

[68] El-Hattab A.W., Scaglia F. (2016). Mitochondrial Cardiomyopathies. Front. Cardiovasc. Med..

[69] Bernier F.P., Boneh A., Dennett X., Chow C.W., Cleary M.A., Thorburn D.R. (2002). Diagnostic criteria for respiratory chain disorders in adults and children. Neurology.

[70] Yang J., Chen S., Duan F., Wang X., Zhang X., Lian B., Kou M., Chiang Z., Li Z., Lian Q. (2022). Mitochondrial Cardiomyopathy: Molecular Epidemiology, Diagnosis, Models, and Therapeutic Management. Cells.

[71] Faienza M.F., Meliota G., Mentino D., Ficarella R., Gentile M., Vairo U., D’amato G. (2024). Cardiac Phenotype and Gene Mutations in RASopathies. Genes.

[72] Hilal N., Chen Z., Chen M.H., Choudhury S. (2023). RASopathies and cardiac manifestations. Front. Cardiovasc. Med..

[73] Delogu A.B., Limongelli G., Versacci P., Adorisio R., Kaski J.P., Blandino R., Maiolo S., Monda E., Putotto C., De Rosa G. (2022). The heart in RASopathies. Am. J. Med Genet. Part C Semin. Med Genet..

[74] Alexandridis G.M., Pagourelias E.D., Fragakis N., Kyriazi M., Vargiami E., Zafeiriou D., Vassilikos V.P. (2022). Neuromuscular diseases and their cardiac manifestations under the spectrum of cardiovascular imaging. Heart Fail. Rev..

[75] Cesar S. (2018). Neuromuscular diseases with hypertrophic cardiomyopathy. Glob. Cardiol. Sci. Pr..

[76] Asatryan B., Rieder M., Murray B., Muller S.A., Tichnell C., Gasperetti A., Carrick R.T., Joseph E., Leung D.G., Riele A.S.T. (2025). Natural History, Phenotype Spectrum, and Clinical Outcomes of Desmin (DES)-Associated Cardiomyopathy. Circ. Genom. Precis. Med..

[77] Clemen C.S., Herrmann H., Strelkov S.V., Schröder R. (2013). Desminopathies: Pathology and mechanisms. Acta Neuropathol..

[78] Hespe S., Ingles J. (2024). Expanding the Phenotypic Spectrum of Desminopathy. JACC Clin. Electrophysiol..

[79] Gerull B., Klaassen S., Brodehl A. (2019). The genetic landscape of cardiomyopathies. Genetic Causes of Cardiac Disease.

